# Modulating the Kynurenine pathway or sequestering toxic 3-hydroxykynurenine protects the retina from light-induced damage in *Drosophila*

**DOI:** 10.1371/journal.pgen.1010644

**Published:** 2023-03-23

**Authors:** Sarita Hebbar, Sofia Traikov, Catrin Hälsig, Elisabeth Knust

**Affiliations:** Max-Planck-Institute of Molecular Cell Biology and Genetics, Dresden, Germany; Instituto Leloir, ARGENTINA

## Abstract

Tissue health is regulated by a myriad of exogenous or endogenous factors. Here we investigated the role of the conserved Kynurenine pathway (KP) in maintaining retinal homeostasis in the context of light stress in *Drosophila melanogaster*. *cinnabar*, *cardinal* and *scarlet* are fly genes that encode different steps in the KP. Along with *white*, these genes are known regulators of brown pigment (ommochrome) biosynthesis. Using *white* as a sensitized genetic background, we show that mutations in *cinnabar*, *cardinal* and *scarlet* differentially modulate light-induced retinal damage. Mass Spectrometric measurements of KP metabolites in flies with different genetic combinations support the notion that increased levels of 3-hydroxykynurenine (3OH-K) and Xanthurenic acid (XA) enhance retinal damage, whereas Kynurenic Acid (KYNA) and Kynurenine (K) are neuro-protective. This conclusion was corroborated by showing that feeding 3OH-K results in enhanced retinal damage, whereas feeding KYNA protects the retina in sensitized genetic backgrounds. Interestingly, the harmful effects of free 3OH-K are diminished by its sub-cellular compartmentalization. Sequestering of 3OH-K enables the quenching of its toxicity through conversion to brown pigment or conjugation to proteins. This work enabled us to decouple the role of these KP genes in ommochrome formation from their role in retinal homeostasis. Additionally, it puts forward new hypotheses on the importance of the balance of KP metabolites and their compartmentalization in disease alleviation.

## Author summary

Intracellular metabolic pathways regulate tissue homeostasis. Less understood are (i) the role of specific metabolic intermediates, and (ii) the interplay between metabolic perturbation and heightened stress. These were addressed in the *D*.*melanogaster* (fly) retina using a light stress paradigm in the context of the conserved Kynurenine pathway (KP). Genes that encode distinct steps of the KP (*cinnabar*, *cardinal* and *scarlet*), along with *white*, are also the genetic regulators of brown pigment biosynthesis in the fly retina. With *white* as a sensitized genetic background, mutations in KP genes differentially modulated light-induced retinal damage. Connecting Mass Spectrometric measurements of KP metabolic intermediates with retinal health showed that increased levels of 3-hydroxykynurenine (3OH-K) and Xanthurenic acid enhanced tissue damage, whereas Kynurenic Acid (KYNA) and Kynurenine are protective. Feeding metabolic intermediates (3OH-K or KYNA) corroborated the above. This has helped to decouple the role of KP genes in pigment formation from their role in retinal homeostasis. Studying these KP genes enabled to better understand the importance of compartmentalization in metabolism. Sequestering of 3OH-K enables the quenching of its toxicity through conversion to brown pigment or conjugation to proteins. In a broad context, it highlights the relevance of compartmentalization of KP metabolites in disease alleviation.

## Introduction

**Fig 1:**
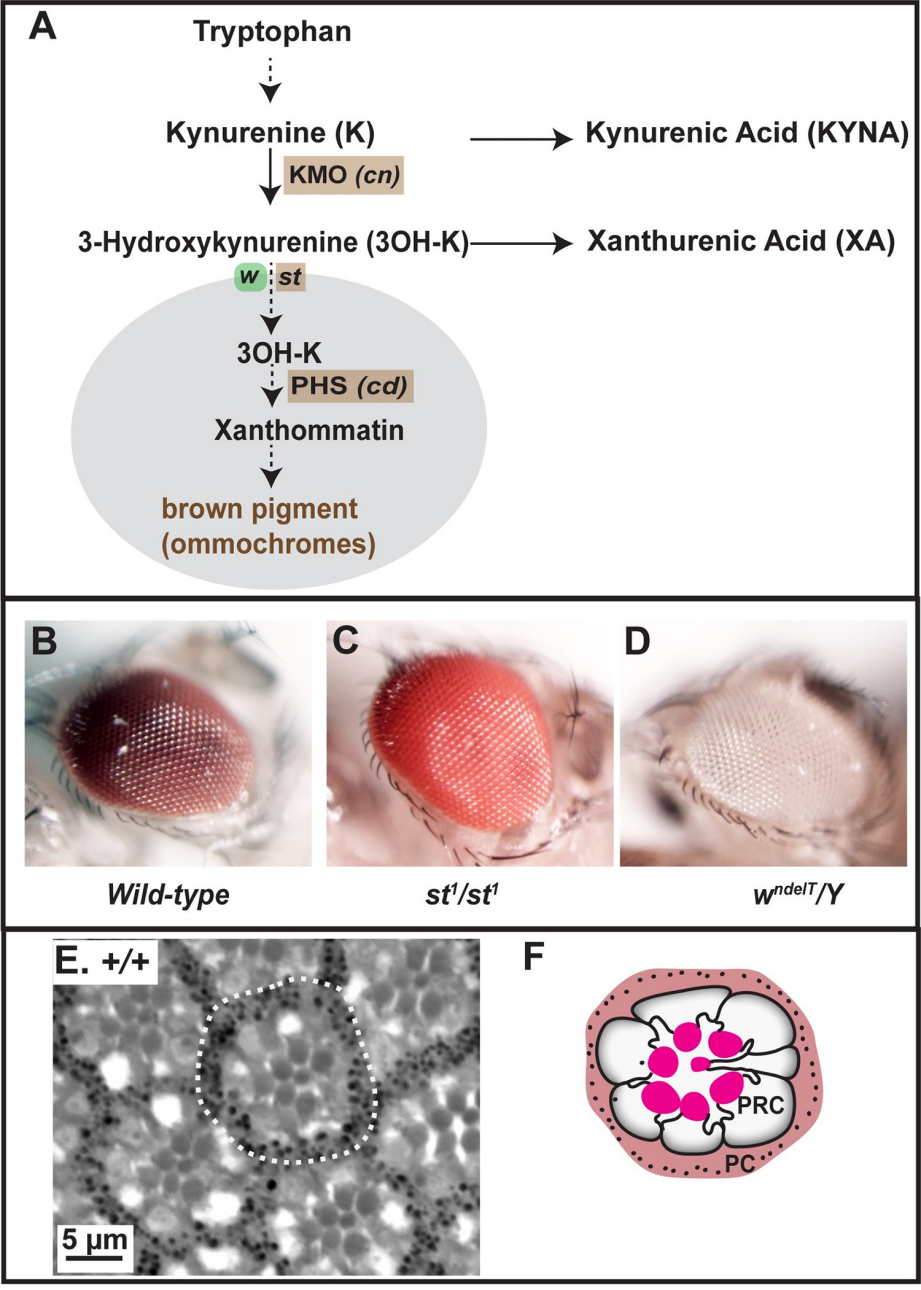
The Kynurenine pathway (KP) pathway in the context of brown pigment biogenesis in the fly eye. **A:** Relevant steps of the KP [from the KEGG database [75]], starting from Tryptophan and leading to brown pigments/ommochromes. Solid arrows: direct steps as precursor-product conversion; dotted arrows: formation of a relevant product after a series of steps. Kynurenine 3-monooxygenase (KMO), and Phenoxazinone synthetase (PHS) are enzymes encoded by the *Drosophila* genes *cinnabar* (*cn*) and *cardinal* (*cd*), respectively. White and Scarlet, encoded by *white* (*w*) and *scarlet* (*st*), respectively, compartmentalize free 3OH-K through their transporter activity, allowing the formation of the brown pigment/ommochromes within lysosome-related organelles (LRO; grey). **B-D:** Photographs depicting eye color phenotypes of the mutants used in this study. (B) Wild type eye (dark red color), (C) bright-red eye color observed in a *st*^*1*^*/st*^*1*^mutant when brown pigment biogenesis is disrupted, (D) non-pigmented, white eye observed when brown and red pigment biogenesis is disrupted as in *a mutant allele of white (w*^*ndelT*^*/Y)*. **E-F**: Bright-field image (E) of a toluidine blue-stained 1μm thick section of a wild-type adult eye after exposure to continuous, high intensity light, showing several ommatidia. One ommatidium is marked with a dotted outline. Note the seven rhabdomeres in the center. (F) Corresponding schematic of the section in E, showing one ommatidium with seven photoreceptor cells (PRC) with their rhabdomeres (bright red), surrounded by pigment cells (PC).

Tissue health is regulated by a myriad of exogenous and endogenous factors, and deregulation of signaling and metabolic pathways have been linked to human diseases. There is increasing evidence that metabolic pathways play crucial roles in the progression of, or protection from, neurodegenerative diseases, including Parkinson’s and Alzheimer’s disease [1] or retinal diseases [2–5]. In particular, the Kynurenine pathway (KP) has more recently attracted much attention. It is one of the pathways downstream of tryptophan (Trp), and its dysregulation can lead to accumulation of intermediates, which can be either toxic or beneficial and thus can exacerbate or ameliorate the phenotypic outcome of neurodegeneration [6]. In short, Trp is converted by tryptophan 2,3 dioxygenase to N-formyl-L-kynurenine, which is, in a second step, hydrolyzed to kynurenine (K) by kynurenine formamidase [7], followed by conversion to 3-hydroxykynurenine (3OH-K), catalyzed by kynurenine 3-monooxygenase (KMO). Kynurenic Acid (KYNA) and Xanthurenic acid (XA) are other metabolites in this pathway, which can be produced from K and 3OH-K [8], respectively (Fig 1A). K and 3OH-K have been associated with degenerative conditions in various animal models and in human diseased tissues [9]. The KP is under consideration as a therapeutic target in neurological disorders [10,11]. A major conundrum, however, is how these metabolites, for example K or 3OH-K, play modulating, sometimes opposing roles on the disease phenotype [12–16]. Other relevant questions in this context include: How do external factors, such as age or environmental stress, intersect in KP modulation of tissue health? What are the underlying cellular conditions for 3OH-K to exert its toxicity? What are the intracellular interactions of 3OH-K with biomolecules? How do these interactions regulate tissue health?

Here, we address some of the above questions using *Drosophila melanogaster* and thus extend previous work done on KP. In *Drosophila melanogaster*, studies on the KP date back to the 1940s when it was shown that mutations in the eye color gene *vermilion* (*v*), which turns the wild-type eye color from dark red (Fig 1B) to bright red (similar to that shown in Fig 1C), affect the conversion of Trp to Kynurenine (K) [17] (Fig 1A). It was later shown that *v* encodes tryptophan 2,3 dioxygenase [18]. Beside *v*, several other eye color mutants resulting in bright red eyes turned out to be part of the KP pathway also: *cinnabar* (*cn*) encodes kynurenine 3-monooxygenase (KMO), which converts K to 3-hydroxykynurenine (3OH-K), *scarlet* (*st*) encodes a transporter of 3OH-K, and *cardinal* (*cd)* encodes a Phenoxazinone synthetase (PHS) (Fig 1A). The eye color phenotype of *v* flies as well flies carrying mutations in *cn* or *st* (light red instead of dark red) is caused by the lack of the brown pigments, the ommochromes, which, together with the red pigments, the drosopterins, form the screening pigments in the *Drosophila* eye [19]. Screening pigments accumulate in lysosome-related organelles (LROs) within pigment cells, support cells that surround the centrally located photoreceptor cells (PRCs; Fig 1E and 1F). These pigments shield PRCs from high intensity light. Formation of the brown pigment called xanthommatin in LROs depends on the transport of 3OH-K into LROs. This is mediated by proteins encoded by *scarlet* (*st*) and *white (w*), genes encoding members of ABC (ATP-binding cassette) transporters [20–22] (Fig 1A). The subsequent action of Phenoxazinone synthetase, encoded by *cardinal* (*cd*), results in the formation of xanthommatin [23], which eventually gets converted to the brown pigment.

Strikingly, beside their role in ommochrome biosynthesis, *Drosophila cn*/KMO and *st* can modulate degeneration in neurons in disease models such as Huntington’s [24,25] and Parkinson’s disease [26]. These data together with the high degree of genetic conservation of disease genes in *Drosophila* support the fly model as a valuable system to unravel the underlying genetic and molecular basis of neurodegenerative diseases [27,28], including retinal degeneration [29].

Both in human and in flies the severity of a mutant phenotype/symptoms in individuals carrying the same mutation can be highly variable, ranging from complete neuronal degeneration to weaker manifestations of the disease phenotype. This could be traced back to the genetic background, which can modulate the severity of a mutant phenotype in flies [30], mice [31] and human [32]. For example, mutations in *Drosophila w*, which lack all pigments and make the eyes white in color (Fig 1D), modulate retinal degeneration in flies expressing the human Tau protein [33]. This suggests that mutations in *w* sensitize the eyes for degeneration. Given that the KP not only participates in brown pigment formation, but is also part of metabolic processes in cells, we hypothesize that the role of *w* in predisposing eyes to degeneration is linked to an imbalance in some KP metabolites, rather than to its role in pigment biosynthesis and hence shielding the eye from excess light. Data presented here using genetic interactions, biochemical analysis of metabolites and dietary intervention support this hypothesis. These approaches enabled us to identify a correlation between higher abundances of 3-hydroxy Kynurenine (3OH-K) under physiological conditions and the severity of retinal damage upon light stress conditions. The causal relationship between metabolite abundance and phenotype could be further documented by showing that dietary supplementation of 3OH-K modulates the degree of light-induced damage. Our results further suggest that free 3OH-K, rather than compartmentalized and protein-bound 3OH-K, is particularly detrimental to the retina.

## Results

### The degree of light-induced damage in eyes of *white* mutant flies is enhanced by mutations in *scarlet*

The *Drosophila* retina is built by about 700 individual units, called ommatidia, which are arranged in a highly stereotypic pattern. Each ommatidium is composed of pigment cells forming a hexagon, which embrace the photoreceptor cells (PRCs). Seven PRCs can be recognized in each section by their rhabdomeres, the photosensitive structure of each cell (Fig 1E and 1F). This regular structure is ideally suited to detect even subtle changes, both in tissue structure and in retinal health (for example by reduced number of PRCs).

**Fig 2:**
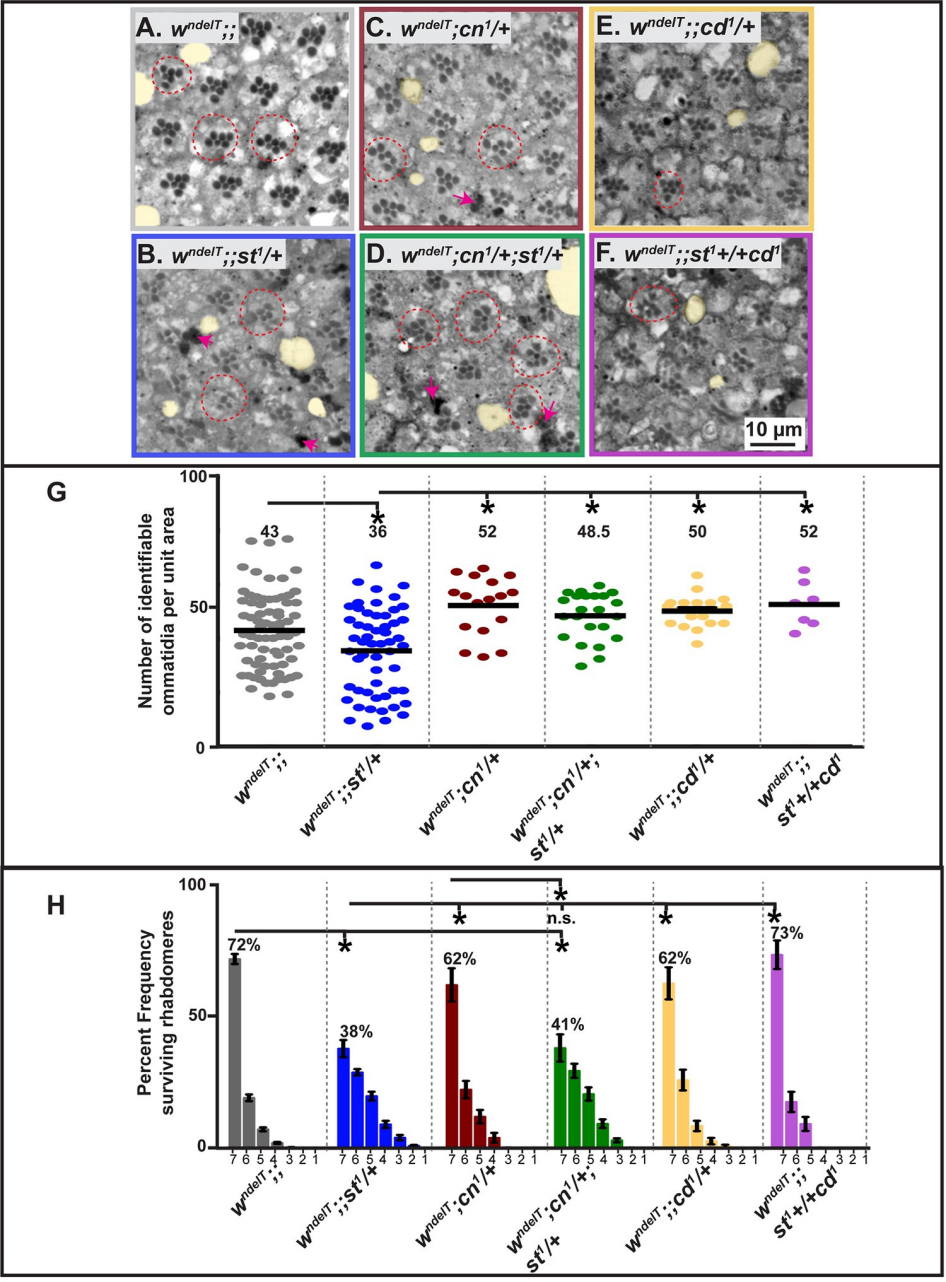
Perturbation of the Kynurenine pathway (KP) modulates retinal damage in *w;;st* mutants. **A-F:** Light microscopic images of toluidine blue-stained, 1μm thin sections of adult fly eyes exposed to continuous, high intensity white light for seven days. Damage attributes are observed such as holes (lacunae) (highlighted in yellow), ommatidia with less than 7 rhabdomeres (red dotted circles), presence of apoptotic debris (intensely stained regions; red arrows). Scale bar as in F. Degenerative attributes appear more pronounced in *w*^*ndelT*^*;;st*^*1*^*/+* (B; severe) and *w*^*ndelT*^*;cn*^*1*^*/+;st*^*1*^*/+* (D; moderate) than in other genotypes. **G:** Graph comparing the extent of damage following exposure of flies of different genotypes to continuous, high intensity white light for 7 days. Horizontal bars indicate the average number of ommatidia with identifiable rhabdomeres of PRCs, normalized to unit area. Each dot represents an individual count from 1 biological replicate. Numbers above each genotype reflect the average for this population. Only statistically significant differences between genotype pairs, as revealed by ANOVA followed by Bonferroni’s post-hoc test at p<0.05, are indicated by *. **H:** Graph representing mean percent frequency [± s.e.m.] of ommatidia displaying 1–7 rhabdomeres following continuous, high intensity white light exposure for 7 days. Numbers above the bars indicate the mean value of ommatidia displaying the full complement of 7 rhabdomeres of each genotype. Statistically significant differences between genotype pairs are indicated by * as revealed by ANOVA followed by Bonferroni’s post-hoc test at p<0.05. “n.s” indicates no statistically significant difference.

Flies carrying mutations in the *Drosophila white* (*w*) gene, which lack eye pigments (Fig 1D), undergo progressive age- and light-dependent retinal degeneration [34,35], which is enhanced by constant illumination [35,36]. To quantify these defects, we defined three phenotypes. (i) Ommatidia with fewer than the normal complement of 7 rhabdomeres (red outlines in Fig 2A–2F). This reflects impaired retinal health, visible at the level of the PRCs. (ii) Density of ommatidia within the tissue. Holes between adjacent ommatidia (highlighted in yellow in Fig 2A–2F) [also called lacunae [34]], cause a disruption in the arrangement of adjacent ommatidia. (iii) Intensely-stained aggregates (red arrowheads in Fig 2B–2D), reminiscent of apoptotic bodies. All three phenotypes are indicative of retinal degeneration and a consequence of exposure to high intensity continuous light. For example, holes and lack of rhabdomeres in *w* mutants are not visible when flies are exposed to 12 hours light/12 hours dark (Hebbar et al., 2017). Quantification of rhabdomere number per ommatidium and density of ommatidial units allows us to compare the degree of retinal degeneration in different genotypes following light-exposure. This quantification enabled us to define a spectrum of light-induced histological changes, ranging from mild damage (with qualitative changes as described above), to moderate degeneration (qualitative changes + significant changes to one of the quantified attributes) to severe (qualitative changes + significant changes to both quantified attributes) degeneration.

In all experiments, young flies (2–4 days) were used. We used *w*^*ndelT*^, a null allele previously designated as w* in [37]. In contrast to wild-type eyes, or to eyes mutant for *w*^*ndelT*^ exposed for the same time to less stringent light conditions, e.g. light exposure interspaced with equal intervals of darkness [37], *w*^*ndelT*^ showed retinal abnormalities upon exposure to continuous, high-intensity light for seven days. First, the retina of light-exposed *w*^*ndelT*^ mutant flies exhibited holes between adjacent ommatidia (Fig 2A). Nevertheless, the number of identifiable ommatidia per unit area (Fig 2G) was not significantly different from that in wild-type retinas (S1A Fig), pointing to largely intact tissue integrity. Second, in *w*^*ndelT*^ mutant retinas, 28% of the ommatidia exhibited less than the full complement of seven rhabdomeres (Fig 2A, red outlines, and 2H). Third, there were hardly any large, intensely-stained aggregates in *w*^*ndelT*^ mutant retinas, reminiscent of apoptotic bodies that have been associated with cell death and degeneration in the retina [38]. Thus, *w*^*ndelT*^ retinas exposed to high intensity light as applied here exhibited mild signs of light-induced damage.

*Drosophila w* encodes an ATP-binding cassette (ABC) transporter of the ABCG subfamily [39,40], which is localized in the membrane of the pigment granule-containing compartment, called the lysosome-related organelle (LRO) [41], and is required to transport 3OH-K from the cytoplasm into the LROs (see Fig 1A). Therefore, we asked whether the mild, light-induced retinal damage in *w*^*ndelT*^ eyes is due to an impaired kynurenine metabolic pathway (KP) rather than to the lack of screening pigments. To address this question, we studied the role of other genes involved in the KP (Fig 1A) on the retinal phenotype of *w*^*ndelT*^, since some metabolic intermediates of this pathway, including 3OH-K, have been implicated in other functions, e.g. oxidative stress- and inflammation-induced damage [9].

Flies homozygous mutant for a null allele of *st*, *st*^*1*^, exhibit bright-red eyes due to the lack of the brown pigments, the ommochromes [22] (Fig 1C). It should be noted that no retinal degeneration was observed in *st*^*1*^*/st*^*1*^ kept under constant light (S1A and S1B Fig), or under light/dark conditions [37]. Flies homozygous mutant for *w* and *st* (*w;; st/st*) have white eyes similar as *w* due to the absence of all screening pigments. Upon removal of one or both functional copies of *st* in a *w*^*ndelT*^ mutant background, all three aspects of retinal damage of *w*^*ndelT*^ mutants were enhanced (Fig 2) resulting in severe retinal degeneration. First, more lacunae in the retina of *w*^*ndelT*^*;;st*^*1*^*/+* were observed (compare Fig 2A with 2B). This goes along with a significant decrease in the number of identifiable ommatidia per unit area (Fig 2G) [37]. Second, compared to control, the number of ommatidia with 7 PRCs per section was further decreased, from 72% in *w*^*ndelT*^ to 38% and 32% in *w*^*ndelT*^*;;st*^*1*^*/+* and *w*^*ndelT*^*;;st*^*1*^*/st*^*1*^, respectively (Figs 2H and S1F). Third, densely stained pyknotic bodies were abundant, pointing to apoptosis (Fig 2B, magenta arrows). Interestingly, there was no difference in the extent of retinal degeneration with the removal of one or both functional copies of *st* (S1E and S1F Fig), thus excluding the role of considerable genetic background-related effects. This was further corroborated with use of other genetic tools to perturb *w* and *st* function. Exacerbation of light-induced damage was also observed when another mutant allele of *w* (*w-RNAi*) was combined with *st*^*1*^ (*w-RNAi;;st*^*1*^/*+)* as compared to *w-RNAi* on its own (S1C and S1D Fig). Finally, we observed no dramatic difference in the degree of light-induced damage between male (*w*^*ndelT*^*/Y;;st*^*1*^/*+*) and female (*w*^*ndelT*^*/w*^*ndelT*^*;st*^*1*^/*+*) flies (S2A and S2B Fig) suggesting no overt sex-specific differences in retinal degeneration.

Continuous, high intensity light (as used in our experiments), in particular its ultraviolet and blue wavelength component, has been associated with increased oxidative stress and stress-related signaling [35,42–44]. Therefore, we speculated that it may be oxidative stress and/or stress-related signaling that contribute to the phenotypes described above. In fact, eliminating these shorter wavelengths of light during the course of the constant light exposure abolished damage to retinal tissue in *w*^*ndelT*^ retinas and in *w*^*ndelT*^*;;st*^*1*^*/+* and *w*^*ndelT*^*;;st*^*1*^*/st*^*1*^ retinas. None of the genotypes showed signs of tissue damage, as revealed by a comparable number of identifiable ommatidia per unit area in all three genotypes (S1E Fig). Moreover, almost all (100%, 80%, 99%) ommatidia exhibited 7 rhabdomeres in *w*^*ndelT*^, *w*^*ndelT*^*;;st*^*1*^*/+* and *w*^*ndelT*^*;;st*^*1*^*/st*^*1*^ eyes, respectively (S1F Fig). Thus, removal of the stress inducing component of white light is sufficient to not only eliminate the exacerbated degeneration in *w;;st* eyes but also to eliminate the basal level of retinal damage in *w* eyes alone.

Taken together, reduction or loss of *st* function enhanced the severity of the *w*^*ndelT*^ retinal phenotype in the presence of exogenous stress, such as high intensity blue light. This severe retinal degeneration is associated with altered pigment cell homeostasis (S3 Fig). In other words, the wild-type function of *st* protects a pigment-less, sensitized *w*^*ndelT*^ retina from degeneration. This relationship of *w* and *st* is not completely unprecedented and has been previously observed in learning behavior [45] where *st* appears to act independently of *w*. Also in the context of tau toxicity [33], each of the half transporters, *white*, *brown*, and *scarlet* have a different epistatic relationship than that seen in pigment biogenesis.

Since *w*^*ndelT*^, *w*^*ndelT*^*;;st*^*1*^*/+*, *w*^*ndelT*^*;;st*^*1*^*/st*^*1*^, *w-RNAi*, and *w-RNAi;;st*^*1*^*/+* all have unpigmented (white) eyes, the lack of screening pigments cannot be the cause for the enhancing role of *st*. This raised the question whether the wild-type metabolic roles of *w*^*+*^ and *st*^*+*^ in trafficking tryptophan metabolites in pigment cells (Fig 1A) [20] protect the retina against light-induced damage.

### Mutations in *cinnabar* (*cn*)/KMO modulate the extent of light-induced retinal damage in *w;;st* double mutants

The neuroprotective role of functional *st*^*+*^ in protecting a pigment-less retina from severe light-induced damages is in line with the observation that *st*^*+*^ prevents neurodegeneration in a fly model for Parkinson’s disease [26]. *st*, together with *w*, is involved in the transport of 3OH-K, an intermediate in the kynurenine pathway, from the cytoplasm into LROs [26,41] (Fig 1A). This led to the suggestion that loss of *st* leads to accumulation of 3OH-K outside the LRO [26,41]. In line with this, we hypothesized that excess cytoplasmic 3OH-K levels are responsible for the enhancing effects on the *w*^*ndelT*^ retinal phenotype upon removal of one copy of *st*. To test this hypothesis, we utilized mutations in *cinnabar* (*cn*), the gene that encodes kynurenine monooxygenase (KMO), an enzyme that catalyzes the formation of 3OH-K from Kynurenine [46–48] (Fig 1A). We expected that reduced levels of 3OH-K by lowering or removing *cn* activity should attenuate the detrimental effects exerted by loss of *st* on the *w*^*ndelT*^ phenotype. In fact, concomitantly removing one copy of *st* and *cn* in a *w* background (*w*^*ndelT*^*;cn*^*1*^*/+; st*^*1*^*/+*) reduced the severity of the tissue damage observed in *w*; *st*^*1*^*/+* retinas. The retinal phenotype of the triple mutant was qualitatively intermediate between the more damaged *w*^*ndelT*^*;;st*^*1*^*/+* and the less damaged control, *w*^*ndelT*^*;cn*^*1*^*/+* (compare Fig 2D with 2B and 2C). Ommatidial density was significantly higher in the triple mutant than in *w*^*ndelT*^*;;st*^*1*^*/+* and very similar to that of *w*^*ndelT*^*;cn*^*1*^*/+* (Fig 2G). In contrast, the percentage of ommatidia with seven rhabdomeres in the triple mutant (41%) was similar to that determined for *w*^*ndelT*^*;;st*^*1*^*/+* (38%) and significantly lower than *w*^*ndelT*^*;cn*^*1*^*/+* (62%) (Fig 2H). Therefore, reducing *cn*/KMO attenuates only the ommatidial density phenotype of a *w*^*ndelT*^*;;st*^*1*^*/+* retina. In contrast, reducing *cn*/KMO does not increase the percentage of surviving rhabdomeres in the triple mutant (Fig 2H). This supports the notion that modulation of light-induced retinal degeneration in these genotypes is reflected primarily in the changes in overall tissue structure (ommatidial density), and the effects on PRCs might be indirect. This phenotype is not allele-specific and could also be observed with *cn*^*35K*^*/+*, a hypomorphic allele (S2C and S2D Fig). Therefore, reducing *cn*/KMO attenuates the phenotype of a *w*^*ndelT*^*;;st*^*1*^*/+* retina suggesting a neuroprotective function in line with its suggested role in the biosynthesis of 3OH-K.

### Mutation in *cardinal* (*cd*)/PHS reduces the extent of light-induced retinal damage in *w;;st* double mutants

To further corroborate the hypothesis that 3OH-K abundance plays a role in light-induced retinal degeneration, we studied *cardinal* (*cd*), a gene downstream of *w* and *st* in the KP pathway. *cd* encodes Phenoxazinone synthetase (PHS), an enzyme that participates in the conversion of 3OH-K to the brown pigment in the LRO [23,49] (Fig 1A). As a result, *cd* mutants have bright red eyes and highly elevated levels of 3OH-K [23,50]. Therefore, we expected an enhancing effect on the *w*^*ndelT*^ retinal phenotype by removing one copy of *cd*, similar to the effects obtained upon removal of one copy of *st*. However, halving the dose of *cd* in a *white* background (*w*^*ndelT*^*;cd*^*1*^*/+*) showed less severe light-induced damages than *w*^*ndelT*^*;;st*^*1*^*/+* retinas (compare Fig 2E with 2B). This was reflected by a higher number of identifiable ommatidia per area unit in *w*^*ndelT*^*;;cd*^*1*^*/+* retinas compared to that of *w*^*ndelT*^*;;st*^*1*^*/+* (50 vs. 36; Fig 2G) and a higher percentage of ommatidia with the full complement of seven PRCs (62% vs. 38%; Fig 2H). All features of the mutant phenotype in the triple mutant *w*^*ndelT*^*;;st*^*1*^*+/+cd*^*1*^ were similar to that of the double mutant *w*^*ndelT*^*;;cd*^*1*^*/+* and significantly better than the phenotype in *w*^*ndelT*^*;;st*^*1*^*/+*.

To summarize, removing one copy of *cd*/PHS in a *w* mutant background does not enhance the severity of the *w* mutant phenotype, which is in contrast to the effects observed upon removing one copy of *st* in a *w* mutant background. This result is puzzling, since mutations in both *st* and *cd*/PHS are predicted to have increased 3OH-K levels [23,26,50,51]. A notable point is that the Cd protein is localized in the lumen of the LRO (Harris et al., 2011; Fig 1F) and hence activity of Cd is compartmentalized. Therefore, in *cd* mutants, 3OH-K likely accumulates in the LRO, thus preventing it from exerting its toxicity. In contrast, St is localized in the membrane of the LRO and in conjunction with White, it transports 3OH-K from the cytoplasm to the LRO (Mackenzie et al., 2000).

Overall our genetic experiments of combining *w*^*ndelT*^*;;st* with either *cn* or *cd* allowed us to speculate that (1) 3OH-K and/or its metabolites can affect retinal health following light exposure in a sensitized background (such as *w*^*ndelT*^), and (2) the detrimental consequences of increased abundance of 3OH-K on the *w* retinal phenotype depend on its sub-cellular localization.

### Mutations in *cn/KMO*, *st* and *cd/PHS* in pigmented eyes have distinct outcomes on the abundances of KP metabolites

To validate the hypothesis that phenotypes described above can be attributed to aberrant levels of 3OH-K and/or its metabolites, we extracted metabolites from the heads of single, double or triple mutants, and identified and quantified them by Mass Spectrometry. We measured Kynurenine (K) and 3-Hydroxykynurenine (3OH-K), the direct metabolites in ommochrome biosynthesis, and Kynurenic Acid (KYNA) and Xanthurenic acid (XA), direct derivatives of K and 3OH-K, respectively (see Fig 1A). It should be noted that the measurement of metabolites from the different mutants was carried out using extracts of heads of flies kept under normal light/dark conditions to preclude any degeneration. Thus, any change in abundance of these metabolites in a particular genotype could potentially be connected to the susceptibility/vulnerability of that genotype to light stress.

The data obtained from the measurements of the metabolites in the different eye color mutants, performed in a pigmented background, are listed in Table 1 and schematized in S4 Fig. Major results can be summarized as follows.

First, removing both copies of either *cn* or *st* in an otherwise wild-type background (red eyes) exhibited no change in the levels of 3OH-K of adult heads (S4A’ Fig, lanes 2 and 3, respectively).

Second *cd* mutants showed significantly higher 3OH-K levels than the wild-type control, as expected (S4A’ Fig, lane 4).

Third, 3OH-K was significantly increased in *st*^*1*^*/st*^*1*^ and *cd*^*1*^*/cd*^*1*^ retinas compared to *cn*^*1*^*/cn*^*1*^ (S4A’ Fig, lanes 2, 4 versus 3 respectively).

Fourth, XA, a metabolite of 3OH-K (see Fig 1A) was significantly higher in *st*^*1*^*/st*^*1*^ mutant retinas, but not in *cd*^*1*^*/cd*^*1*^ retinas, when compared to wild-type (S4B’ Fig, lanes 2, 4 versus lane 1).

Fifth, no significant changes were observed for K and KYNA in the genotypes examined when compared to wild-type (Table 1 and S4C and S4D’ Fig).

**Fig 3:**
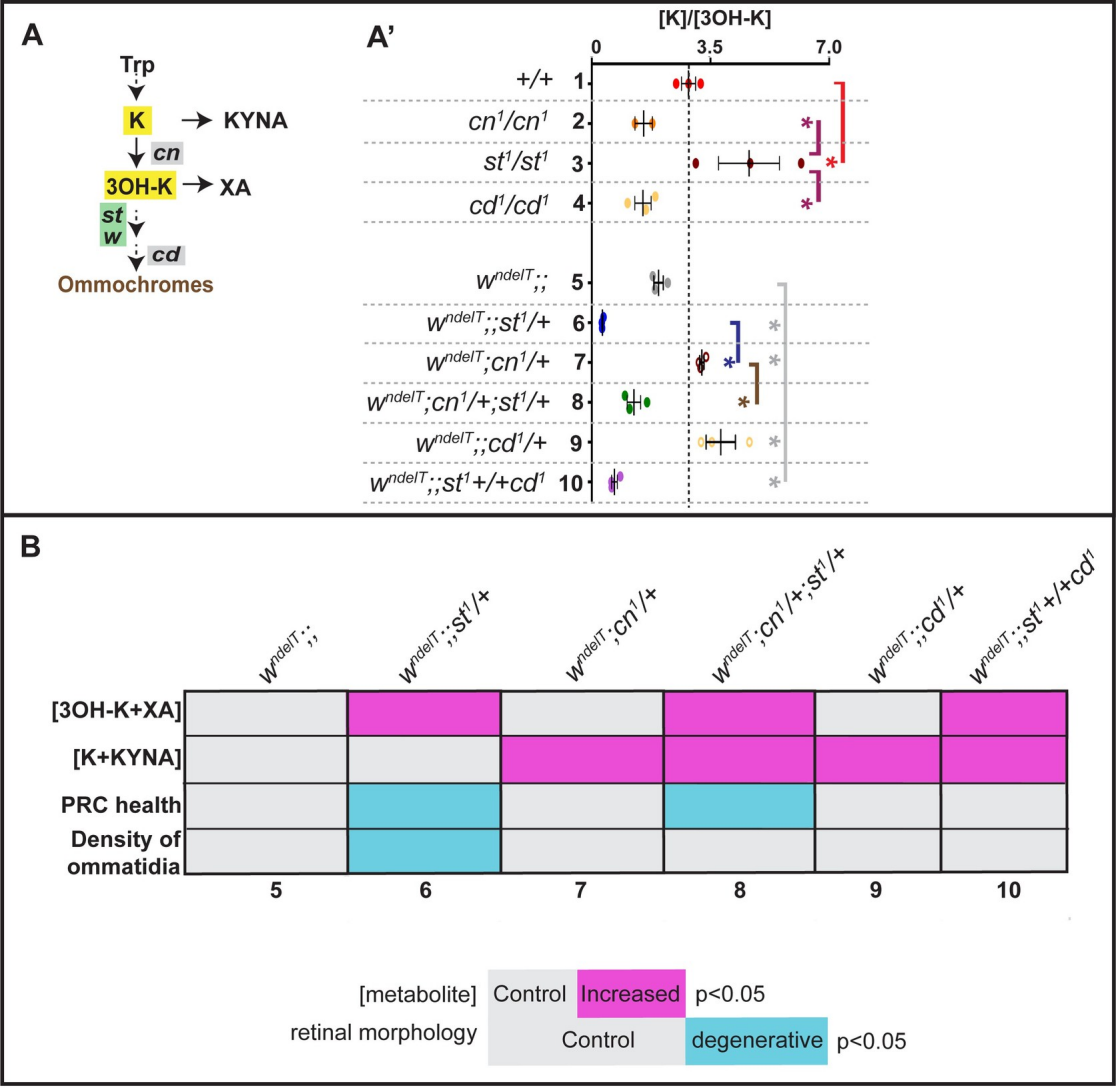
Increased 3OH-K abundance is associated with extensive light-induced retinal damage. **A:** Schematic outline of relevant steps in the Kynurenine pathway (KP) and brown pigment biosynthesis. Genes used in this study are indicated. **A’:** Graph depicting the ratio of the abundances of Kynurenine and 3-hydroxykynurenine [K]/[3OH-K] (highlighted in yellow in the corresponding pathway shown in A-D) from MS measurements of head extracts of flies kept under physiological/non degenerative light conditions. Numbers on Y-axis correspond to the genotypes listed to the left. Each colored dot represents an individual biological replicate of 10 pooled heads. Statistical comparisons between genotype pairs are indicated with solid lines (red line compared to *+/+*, magenta line compared to *cn*^*1*^*/cn*^*1*^, grey line compared to *w*^*ndelT*^, blue line compared to *w*^*ndelT*^*;;st*^*1*^*/+*, brown line compared to *w*^*ndelT*^*;cn*^*1*^*/+*) with * revealed by ANOVA followed by Bonferroni’s post-hoc test at p<0.05. **B:** Tabular summary of various parameters (horizontal lines) with different genotypes (columns 5–10, corresponding to genotypes shown in the table in A’). The parameters include metabolite levels (top two) and retinal health indicators (bottom two). For metabolite levels, grey colored cells imply no difference from controls (*w*^*ndelT*^), cells colored magenta imply significantly higher metabolite levels. For retinal health indicators, grey colored cells imply no difference from controls (*w*^*ndelT*^), cells colored cyan imply significantly reduced ommatidial density (number of identifiable ommatidia per unit area) or PRC health (percent frequency of surviving rhabdomeres). Statistical significances were calculated with ANOVA followed by Bonferroni’s post-hoc test at p<0.05.

Fig 3A’ illustrates the results by showing the ratio of precursor/product, i.e. [K]/[3OH-K]. In agreement with data from another allele, *cn*^*3*^ [24,52,53], and as expected from the lack of an enzyme (KMO) that converts K to 3OH-K, this ratio is significantly increased in *cn*^*1*^ mutant eyes. On the other hand, no change in ([K]/[3OH-K]) was observed in *st* mutants. This is consistent with previous reports, which explained the lack of increased 3OH-K in heads of *st* mutant adults by its excretion via Malpighian tubules at the end of pupal development [23,51]. Interestingly this elimination of 3OH-K is not observed in *cd*/PHS mutants [also in [54]], suggesting that here compartmentalization and/or another alternate mechanism sequesters 3OH-K.

**Table 1:** Metabolite abundance across genotypes.

Genotype	Metabolite			
	3OH-K	XA	K	KYNA
Pigmented Eyes				
*+/+*	103.7±2.2	1124±141.5	294.9±25.2	36.2±1.1
*st* ^*1*^ */st* ^*1*^	148.4±2.4	28351±556*	226.5±34.2	83.3±7.5
*cn* ^*1*^ */cn* ^*1*^	90.1±2.2	1891±115.6	414.0±71.7	83.9±8.5
*cd* ^*1*^ */cd* ^*1*^	165.1±11.7*	446.1±115.8	243.6±23.5	87.8±4.3
Non-pigmented Eyes				
*w* ^*ndelT*^	87.9±8.5	1079±52	174.0±24	82.1±3.1
*w* ^*ndelT*^ *;;st* ^*1*^ */+*	236.2±11.9†	29854±3771†	74.06±0.7	120.3±13.3
*w* ^*ndelT*^ *;cn* ^*1*^ */+*	133.9±4.3	4794±761.9	433.8±6.3†	561.3±21.2†
*w* ^*ndelT*^ *;cn* ^*1*^ */+;st* ^*1*^ */+*	204.9±27.7†	19321±3191†	248.1±30.3	624.3±51.5†
*w* ^*ndelT*^ *;;cd* ^*1*^ */+*	112.4±3.2	4511±693	429.3±58.5†	734.1±30.2†
*w* ^*ndelT*^ *;; st* ^*1*^ *+/+cd* ^*1*^	350.1±246†	52447±4428†	232.3±18.2	451.9±40.6†

Values are mean ± s.e.m (in arbitrary units) for the abundances of metabolites (sample size = 3 biological replicates)

Statistical significance following ANOVA and post-hoc Bonferroni’s test denoted by

*p<0.05 (compared to *+/+*)

†p<0.05 (as compared to *w*^*ndelT*^)

K: Kynurenine

KYNA: Kynurenic Acid

3OH-K: 3-Hydoxykynurenine

XA: Xanthurenic Acid

In conclusion, our measurements of KP metabolites in pigmented eyes provide a biochemical characterization of the KP as shown in Fig 1A. Further, these data support the idea that mutations in *st* and *cd*/PHS have different outcomes on the KP.

### Increased abundance of 3OH-K in *w;;st* mutants is associated with increased susceptibility to retinal damage

Next, we determined the effects of removing single copies of genes of the KP (*cn*/KMO, *st*, *cd*/PHS) on the different KP metabolites in a *w* mutant background, to correlate with changes in mutant phenotype.

First, *w*^*ndelT*^ on its own displayed no significant changes in any of the metabolites when compared to +/+ (Table 1 and S4 Fig).

Second, significantly higher 3OH-K and XA levels compared to *w*^*ndelT*^ were observed in the double mutant *w*^*ndelT*^*;;st*^*1*^*/+* and in the triple mutants *w*^*ndelT*^*;cn*^*1*^*/+; st*^*1*^*/+* and *w*^*ndelT*^*;;st*^*1*^ *+/ + cd*^*1*^ (Table 1 and S4A’ and S4B’ Fig, lane 6 versus lane 5, lane 8 versus lane 5, and lane 10 versus lane 5, respectively).

Third, K and KYNA abundances were unaltered in *w*^*ndelT*^*;;st*^*1*^*/+* mutant as compared to *w*^*ndelT*^ (S4C’ and S4D’ Fig, lane 6 versus lane 5).

Fourth, significantly higher levels of K and KYNA were detected in *w*^*ndelT*^*;cn*^*1*^*/+* and *w*^*ndelT*^*;cn*^*1*^*/+;st*^*1*^*/+* compared to *w*^*ndelT*^ and *w*^*ndelT*^*;;st*^*1*^*/+*, respectively (S4C’ and S4D’ Fig, lane 7 versus lane 5, and lane 8 versus lanes 6, respectively).

Fifth, a significant increase in KYNA levels was observed in *w*^*ndelT*^*;;cd*^*1*^*/+* and *w*^*ndelT*^*;;st*^*1*^*+/+cd*^*1*^ as compared to *w*^*ndelT*^ (Table 1 and S4D’ Fig, lane 9 versus lane 5 and lane 10 versus lane 5).

Overall, these measurements corroborate the idea that the KP is sensitized by mutations in the *w* gene. This is further demonstrated by examining the ratio of K/3OH-K (Fig 3A). Reducing one component of the KP (either *cn*/KMO, or *st*) in a *w* genetic background results in more pronounced changes in the ratio of K/3OH-K as opposed to its reduction in a wild-type background alone (Fig 3A: in the case of *cn*, lane 1 versus lane 3 and lane 5 versus 7 and in the case of *st*, lane 1versus lane 2 and lane 5 versus lane 6).

Do these metabolite abundances (measured in physiological conditions) correlate with the susceptibility of the retina to light exposure? To better visualize the associations of altered metabolite abundances with retinal health, we designed a color-coded table (Fig 3B), which revealed important trends.

First, increased 3OH-K+XA abundance is linked with severe and moderate retinal phenotypes, as in *w*^*ndelT*^*;;st*^*1*^*/+* and *w*^*ndelT*^*;cn*^*1*^*/+;st*^*1*^*/+*, respectively (Fig 3B, columns 6 and 8). Accordingly, a healthy retina correlates with no changes in 3OH+XA abundance (Fig 3B, columns 5, 7, 9).

Second, the moderate retinal phenotype in *w*^*ndelT*^*;cn*^*1*^*/+;st*^*1*^*/+* despite increased 3OH-K+XA can be explained by concomitant increased K+KYNA abundance (Fig 3B, column 8), which most likely counteracts the detrimental effects of increased levels of 3OH-K and XA. However, differences in the extent of the rescue between *w*^*ndelT*^*;cn*^*1*^*/+;st*^*1*^*/+* and *w*^*ndelT*^*;;st*^*1*^ *+/ + cd*^*1*^ cannot be explained by these metabolite abundances alone (Fig 3B, column 8 versus 10). The moderate degeneration in case of *w*^*ndelT*^*;cn*^*1*^*/+;st*^*1*^*/+* might stem from a separate role of *cn*/KMO in mitochondrial regulation [52] independent of metabolite levels.

In conclusion, these results connect increased levels of KYNA and K with better retinal health, most likely by counteracting the detrimental effects of increased levels of 3OH-K and XA.

### Feeding 3OH-K is sufficient to enhance light-induced retinal damage in a *w*^*ndelT*^ sensitized genetic background

**Fig 4:**
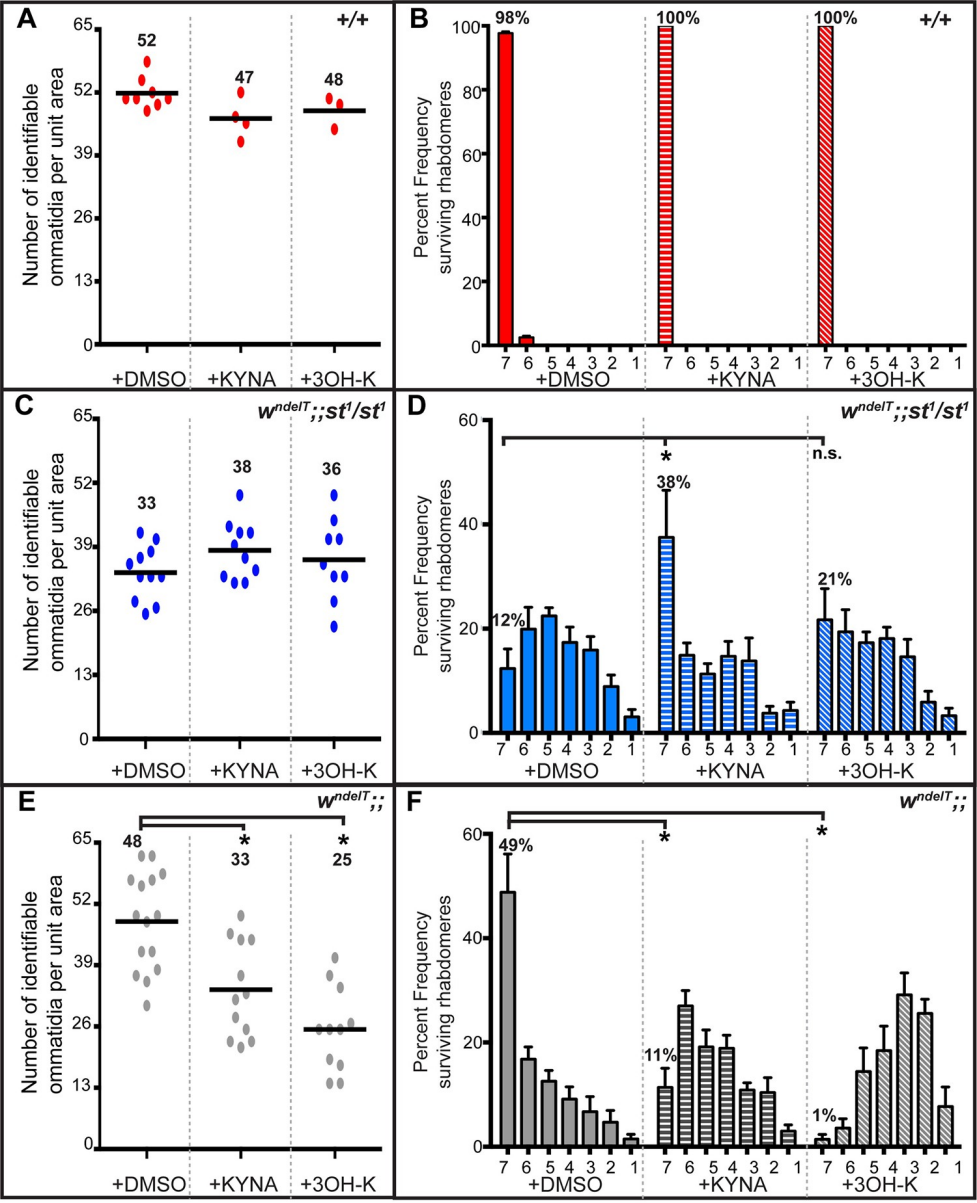
Effect of light-induced damage upon feeding 3OH-K or KYNA to flies of different genotypes in sensitized genetic backgrounds. **A, C, E.** Graphs comparing the extent of retinal damage following exposure to continuous white light in +/+ (red; A), *w*^*ndelT*^*;;st*^*1*^*/ st*^*1*^ (blue; C) and *w*^*ndelT*^ (grey; E) flies raised on control food (+DMSO) or food supplemented with either 1mg/ml KYNA or 1mg/ml 3OH-K. Horizontal bars indicate the average number of identifiable ommatidia normalized to unit area. Each dot represents an individual count from 1 biological replicate. Number indicates the mean value for each condition. Statistically significant differences between genotype pairs are indicated by * as revealed by ANOVA followed by Bonferroni’s post-hoc test at p<0.05. **B, D, F.** Mean percent frequency of ommatidia displaying 1–7 rhabdomeres [± s.e.m.] upon exposure of flies to continuous, high intensity white light for 7 days. *+/+* (red; B), *w*^*ndelT*^*;;st*^*1*^*/ st*^*1*^ (blue; D) and *w*^*ndelT*^ (grey; F) flies were raised on control food (+DMSO) or on food supplemented with either 1mg/ml KYNA (bars with horizontal lines) or 1mg/ml 3OH-K (bars with slanted lines). Numbers above the bars indicates the mean value of ommatidia displaying the full complement of 7 rhabdomeres. * indicates statistical significance for the pair as revealed by ANOVA followed by Bonferroni’s post-hoc test at p<0.05. “n.s” indicates no statistically significant difference.

Our data suggest that 3OH-K metabolites are “toxic” and sensitize the *w*^*ndelT*^ retinal tissue to light-induced damage (as in *w*^*ndelT*^*;;st*^*1*^*/+*), while KYNA exerts a “protective” function (as in *w*^*ndelT*^*;cn*^*1*^*/+;st*^*1*^*/+*). To further corroborate this assumption, we fed animals with “protective” KYNA (1mg/ml) or “toxic” 3OH-K (1mg/ml) and evaluated retinal health upon light exposure. Similar amount of these metabolites has been previously used to demonstrate their importance in neurodegenerative mutants [24]. The feeding regimen used here was sufficiently effective for the incorporation of the metabolites into eyes. For example, feeding 3OH-K (1mg/ml) to *cn*^*1*^*/ cn*^*1*^ mutants, which cannot synthesize 3OH-K and hence develop bright-red eyes due to an impaired ommochrome pathway, was sufficient to restore the complete brown pigment biogenesis pathway and change the eye color to wild-type (S5A Fig). This treatment had no consequences on retinal morphology following light exposure (Fig 4A and 4B), consistent with our earlier observations that KP disruption does not impair retinal morphology upon light exposure in the wild-type background/pigmented eyes.

In contrast, feeding “protective” KYNA to *w*^*ndelT*^*;;st*^*1*^/*st*^*1*^ animals reversed PRC damage observed in control animals (fed with DMSO). 38% of the ommatidia of flies fed with KYNA showed seven rhabdomeres compared to 12% observed in the feeding control (Fig 4D). Thus, feeding KYNA phenocopied the triple mutant *w*^*ndelT*^*;cn*^*1*^*/+; st/+*^*1*^. However, this treatment did not significantly increase the number of identifiable ommatidia per unit area (a measure for tissue integrity) (Fig 4C). Feeding “toxic” 3OH-K to *w*^*ndelT*^*;;st*^*1*^/*st*^*1*^ flies did not further exacerbate light-induced retinal damage in terms of ommatidial integrity (Fig 4C) and PRC damage (Fig 4D). In contrast, feeding 3OH-K in another sensitized background (*w*^*ndelT*^) resulted in significantly more damage compared to the feeding control (with DMSO). The number of identifiable ommatidia per unit area decreased from 48 to 25 (Fig 4E) and the percentage of ommatidia containing seven rhabdomeres was significantly reduced upon feeding 3OH-K, from 49% in the control to 1% (Fig 4F). Finally, in contrast to our expectation, feeding *w*^*ndelT*^ animals with KYNA increased the severity of both read-outs of retinal integrity (Fig 4E and 4F).

Taken together, dietary increase of KYNA protected the *w*^*ndelT*^*;;st*^*1*^/*st*^*1*^ retina from light-induced PRC damage, and hence provides a molecular explanation for the phenotype of the triple mutant. 3OH-K feeding, on the other hand, enhanced the phenotype of *w*^*ndelT*^ flies, thus phenocopying *w*^*ndelT*^*;;st*^*1*^*/+* mutant retinas (Fig 2), which have increased 3OH-K levels (Table 1). Overall, these data strengthen our earlier conclusions that increased 3OH-K (or increased 3OH-K +XA) makes a retina more susceptible to light-damage. On the other hand, increased KYNA (or increased K + KYNA) is protective in a sensitized background.

### Free 3OH-K, but not protein-bound 3OH-K, is connected to susceptibility to light-induced degeneration

**Fig 5:**
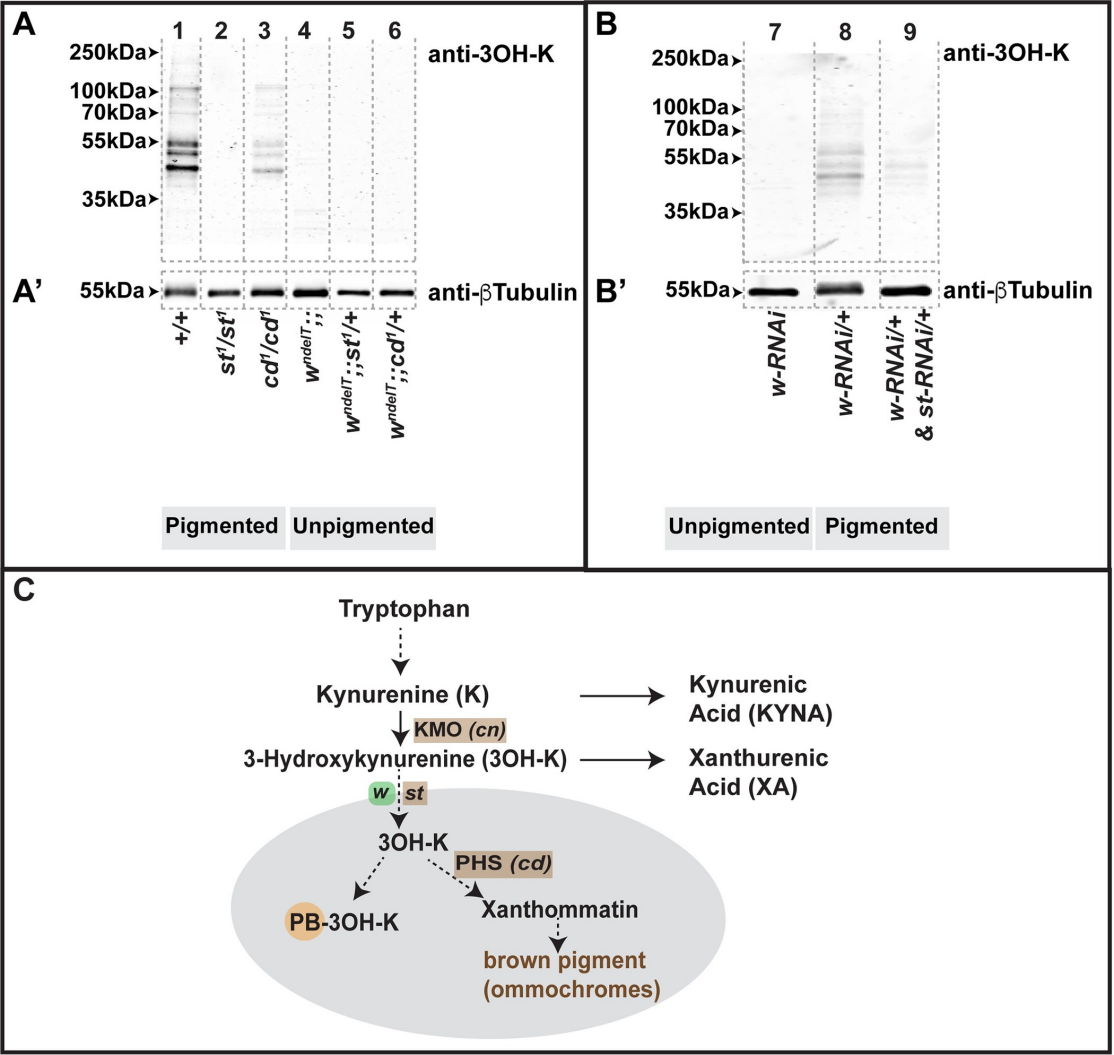
Perturbation of the Kynurenine pathway affects the amount of protein-bound 3OH-K. **A-B’:** Western blots of protein extracts from heads of flies of different genotypes raised on normal food under physiological/non-degenerative conditions. Blots were tested with antibodies against 3OH-K (A, B) and ß-Tubulin as loading control, run on parallel gels (A’, B’). Numbers on the top of the lanes correspond to the genotypes listed below. Positions of standard molecular weight markers are indicated by arrowheads. Vertical, grey dotted lines outline the lanes. **C:** A cartoon depicting selected steps of the Kynurenine pathway. Kynurenine 3-monooxygenase (KMO) and Phenoxazinone synthetase (PHS) are enzymes encoded by *cinnabar* (*cn*) and *cardinal* (*cd*), respectively. White and Scarlet, encoded by *white* (*w*) and *scarlet* (*st*), respectively, compartmentalize free 3OH-K through their transporter activity into LROs (grey). The schematic represents the emerging hypothesis from the experiments shown in A-B’ that 3OH-K can be bound to proteins (PB-3OH-K) only following its transport into LROs by White and Scarlet.

Although both 3OH-K and K+KYNA levels are increased in in *w*^*ndelT*^*;cn*^*1*^*/+;st*^*1*^*/+* and in *w*^*ndelT*^*;;st*^*1*^ *+/+ cd*^*1*^, only the former shows a mutant retinal phenotype (compare column 4 and 6 in Fig 3). This raised the question of the status of free and protein-bound 3OH-K (PB-3OH-K) in the context of the genotypes studied here, since 3OH-K can exist in a protein-bound form in flies [50] and in humans [55]. To test this, we determined the levels of PB-3OH-K in head lysates of flies raised in non-degenerative light conditions, both in wild-type (+/+) and in *w*^*ndelT*^ mutant background. PB-3OH-K was detected in head lysates of *+/+*, and, in lower amounts, in head lysates of *cd*^*1*^*/cd*^*1*^ flies (Fig 5A, lane 1 versus lane 3) as reported previously [50]. Increased PB-3OH-K was also detected in *+/+* flies fed with 3OH-K (S5B Fig). In stark contrast, PB-3OH-K was not detected in head lysates of *st*^*1*^*/st*^*1*^(Fig 5A, lane 2), nor in all genotypes with *w* in the background, namely *w*^*ndelT*^, *w*^*ndelT*^*;;st*^*1*^/+, and *w*^*ndelT*^*;;cd*^*1*^/+ (Fig 5A, lanes 4–6 respectively) and *wRNAi* (Fig 5B, lane 7). These results suggest that 3OH-K is bound to proteins only upon its transport into LROs (Fig 5C) by functional White/Scarlet [41]. PB-3OH-K was also decreased upon the concomitant knockdown of *st* and *w* by RNAi (Fig 5B lane 9) as opposed to knockdown of *w* alone (Fig 5B, lane 8).

On the other hand, in *cd* mutants, free 3OH-K is still transported into LROs, but cannot been utilized for the formation of brown pigment (Fig 5C). However, it is sequestered by conjugation with proteins as evidenced by PB-3OH-K in head lysates of *cd*^*1*^*/cd*^*1*^ (Fig 5A’, lane 3). Further, it should be noted that PB-3OH-K was only detected in wild-type and *cd*^*1*^*/cd*^*1*^, genotypes, both of which do not exhibit retinal damage when exposed to light (S1A and S1A’ Fig). But why do *st* mutant eyes, with no visible PB-3OH-K levels, not exhibit light-induced damage? Residual red pigmentation in *st* mutants absorbs light and shields the retina from high intensity light. When this residual pigmentation is removed as in *w* mutants, which also by themselves do not show increased PB-3OH-K, high intensity light induces retinal damage. Thus, the compartmentalization and conjugation of 3OH-K to proteins suggests a mechanism allowing to sequester and subsequently “detoxify” the effects of free 3OH-K.

Overall, data shown here allow the conclusion that increase of free 3OH-K is detrimental for retinal health in *Drosophila*, in particular in a sensitized background (*w*). More importantly, they unravel the importance of compartmentalization of the KP metabolites as a protective mechanism achieved by sequestering 3OH-K, either via the formation of xanthommatin in LROs by *cd*/PHS, or via the conjugation of free 3OH-K to proteins.

## Discussion

Results presented here underscore the importance of metabolites of the Kynurenine pathway (KP), one of the catabolic pathways for tryptophan, in neurodegeneration. Through the use of a series of fly mutants that perturb distinct steps of the KP (Fig 5C), metabolite analysis, and dietary supplementation, we propose two major mechanisms by which the net outcome of the KP modulates retinal degeneration. First, although increased 3OH-K levels can be detrimental, more important is the balance between toxic 3OH-K and protective KYNA that impacts on the degree of degeneration. In other words, modulating the balance of KP metabolites in favour of increased KYNA protects the retina. Second, 3OH-K only worsens light-induced retinal degeneration when present as free 3OH-K, but not in its protein-bound form (PB-3OH-K). This study now brings to the forefront this previously underestimated aspect of the KP in the context of tissue health. Sequestering of 3OH-K, in protein-bound form or by compartmentalisation, represents a mechanism to mitigate its toxicity in neurodegeneration. This becomes relevant when considering the KP as a therapeutic target in neurological disorders [10,11].

The toxicity of 3OH-K stems from its ability to act as a pro-oxidant that auto-oxidizes (non-enzymatic) itself, followed by oxidation of other proteins or cellular DNA [reviewed [56]]. On the other hand, 3OH-K was shown in different contexts to act as a potent scavenger of radicals, such as reactive oxygen species (ROS). The non-enzymatic oxidation of 3OH-K leads to formation of hydroxanthommatin radicals, which are then dimerized to form xanthommatin [49,57]. Hence, 3OH-K has the potential to act both as an oxidant, producing more ROS, and as antioxidant, depending on its concentration and the overall oxidizing environment in cells [58]. The link of 3OH-K and ROS is particularly important in neuronal cells, including those in the brain and the retina, which are metabolically very active and therefore more vulnerable to hyperoxidation. Evidence that increased 3OH-K exacerbates ROS production also comes from previous observations that brains of *st* mutants have elevated ROS levels (Cunningham et al., 2008) and from our observations of altered oxidative stress signaling in *st* mutants (S6A Fig). Preliminary data (S6B and S6C) allowed us to speculate that a similar increase of ROS in the sensitized *w; st/+* retina makes this tissue more prone to degeneration under light stress. This assumption is supported by the observation that degeneration only takes place upon exposure to high intensity light, in particular light containing UV- and blue-wavelengths (this study). These shorter wavelengths can enhance ROS production [59] and result in increased oxidative stress response [42]. Hence, flies with underlying genetic perturbation of the KP leading to increased 3OH-K are more vulnerable to retinal degeneration when exposed to increased ROS-producing conditions such as high intensity continuous light.

Apart from ROS production, the KP affects many other cellular processes. For example, KP metabolites are regulators of zinc storage in *Drosophila* Malpighian tubules [60]. KP genes such as *cn*/KMO also play a role in mitochondrial dynamics, independent of metabolite abundances [52]. Likewise, retinal health and homeostasis depend on several factors/processes, dysregulation of which, including defects in rhodopsin [61], lipid homeostasis [62–65] and iron storage [66] to name a few, have been implicated in retinal degeneration. Future analyses are necessary to unravel interactions between these cellular processes and the KP in retinal homoestasis.

Results presented here provoke two important questions:

First, *why does a white (w) genetic background influence KP metabolite abundances differently in cn*, *st*, *or cd mutants*? This is puzzling because loss of functional *w*, on its own, does not exhibit any significant changes in KP metabolites. Yet, *w* in combination with *cn*, *st* or *cd* mutants, results in pronounced changes to KP metabolite abundances, more than when *cn*, *st* or *cd* mutants are in a wild-type background. Unexpected changes in KP metabolites due to a *w* genetic background are, however, not unique to our study, and were also observed upon modifying the dosage of *w* [24]. Since measurements as presented here only depict a snapshot of the abundances of these KP metabolites, we cannot exclude an indirect influence as a result of loss of *whit*e on the turnover of KP metabolites in these genotypes. These indirect effects could, for example, stem from the role of White in the import of the precursor Tryptophan [67], or in its role in the metabolism of tetrahydrofolate [53] or sphingolipid degradation [68].

Second, *why does increased 3OH-K not always aggravate the retinal phenotype in a sensitized w background*? Fly models for Parkinson’s disease [26], Huntington’s disease [HD; [24]] and retinal degeneration (this work) reinforce a previously reported connection between neurodegeneration and an imbalance in toxic and protective KP metabolites [69–71]. These models also allowed to establish a causal relationship between an imbalance of metabolites of the KP and the severity of a mutant phenotype. Therefore, the consequence of a disrupted KP in the context of neuronal degeneration should not only take into account changes in the absolute amounts of individual metabolites, but also any imbalances between toxic (3OH-K and XA) and protective (KYNA and K) metabolites as well as their possible conversion to non-toxic moieties (PB-3OH-K). For example, removing one copy of *st* in a *w* mutant background results in elevated 3OH-K levels and severe retinal degeneration. Removing one copy of *cd*/PHS in this background does not affect retinal health despite similarly increased 3OH-K levels, due to concomitant increased K+KYNA abundance. This suggests that increased KYNA levels (reported here) counterbalance the increase in 3OH-K. Elevated KYNA can be protective since it has the potential to neutralize ROS and reduce the oxidative stress burden [72]. Indeed, we could show that feeding KYNA in a sensitized (*w;;st*) background reduces the severity of degeneration. Future studies on the turnover of KP metabolites will help to better understand the upregulation of KYNA in mutant combinations of *w* with *cn*/KMO or *cd*/PHS.

Another mechanism that might explain the lack of toxicity of increased 3OH-K, which have not been considered previously in the fly models, is sequestering of 3OH-K by its conjugation to proteins. This process depends on the transport of 3OH-K into LROs by the W/St transporters (Fig 5C). *cd*/PHS mutants (in a *w*^+^ background) display increased 3OH-K levels. Although *cd* mutants are defective in conversion of free 3OH-K to xanthommatin, they nevertheless form PB-3OH-K, though in lower amounts, thus reducing the detrimental effects of free 3OH-K. This result could also explain a recently published observation that *cd*/PHS mutants show age-related deficits in courtship songs and memory [50]. Furthermore, it might help clarify the contradictory data connecting increased abundance of 3OH-K to neurodegeneration in some studies, and to survival in others.

While conjugation of 3OH-K to proteins reduces free, toxic 3OH-K, at the same time this modification can alter the function of proteins and can convert them to harmful variants. For example, 3OH-K conjugation to Aβ peptides and peptides derived from α-synuclein was suggested to be related to neurodegeneration [73]. In contrast, 3OH-K conjugated to the human eye lens protein [74] was detected in normal aged human lenses but not in damaged (cataract) lenses [55]. In this case, 3OH-K-based protein modifications may actually protect the retina from UV-light-induced damage, and is probably not the cause of age-related eye damage [55]. Data obtained here in the fly retina also support the notion that PB-3OH-K is not harmful, but rather quenches free toxic 3OH-K. Formation of PB-3OH-K is common to conditions that do not display any light-induced degeneration, including wild-type, *cd*/PHS mutants, and wild-type flies fed with 3OH-K.

In contrast, is the absence of PB-3OH-K associated with increased susceptibility to retinal damage under conditions of light stress? This is indeed the case for mutations in the ABCG transporter/*w*. Mutations in *w*, or *w* in any genetic combination studied here, displayed almost no PB-3OH-K and were indeed susceptible to retinal damage of varying severities following light exposure. This suggests that free 3OH-K can be toxic when not converted to xanthommatin or conjugated to proteins. This non-sequestered/free 3OH-K, albeit in the physiological abundance range, might underlie the mild phenotypes in *w* retinas when exposed to light, and hence constitutes a sensitized genetic background. In this context, it should be noted that PB-3OH-K is also absent in *st* retinas, resulting in non-sequestered 3OH-K, yet *st* retinas in a wild-type background do not degenerate. When exposed to high intensity light, the *st* retina is presumably shielded from intense light exposure due to the presence of the red shielding piments. The complete absence of red and brown pigments in *w* therefore make it a more susceptible background when compared to *st* in our study.

In the future, conjugation of 3OH-K to proteins may be considered as a potential therapeutic strategy to be tested in case of other neurodegenerative diseases with KP disruption. It would also be relevant to understand the process and sub-cellular location in which the covalent addition of 3OH-K to proteins occurs. PB-3OH-K was detected by our method in head extracts of *cd*/PHS mutants but not in *w* or *st* mutants. This revealed that 3OH-K conjugation to proteins occurs only once it is compartmentalized into LROs (Fig 5C). In vitro experiments have indicated that 3OH-K is most likely attached to cysteine residues of proteins [55]. However, the identity of the target proteins and the functional consequence of their modification still remain elusive.

Put together, this work highlights a novel function for genes involved in the KP in regulating *Drosophila* retinal health, which is uncoupled from their role in ommochrome formation. The results underscore the importance of compartmentalization of a KP metabolite, 3OH-K, and its quenching to non-toxic PB-3OH-K, in regulating tissue homeostasis.

## Materials and methods

### Fly strains and genetics

All phenotypic analyses were performed in age-matched (2 days of adulthood) males, unless otherwise specified, during the light-cycle of the incubator. Flies were maintained at 25°C on standard food (In water: corn flour-8%, malt-8%, sugar beet molasses-2.2%, soy-1%, yeast-1.8%, agar-0.75%, propionic acid-0.625%, 30% Nipagin (in ethanol)-0.5%). Wild-type flies were from the *Oregon R* strain. The *white* allele, designated as *w*^*ndelT*^*;; or w*^*ndelT*^, as it is a null allele that carries a deletion removing the transcriptional and translation start site of the *w* gene [37]. Other mutant lines were obtained from The Bloomington *Drosophila* Stock Center and include: *cn*^*1*^ (Line ID: BDSC_263), *cn*^*35k*^ (Line ID: BDSC_268), *st*^*1*^ (Line ID: BDSC_605) and *cd*^*1*^ (Line ID: BDSC_3052). Double mutant heterozygous combinations were made by crossing female flies (*w*^*ndelT*^) with males of each of the mutant lines and selecting age-matched male flies. A homozygous line bearing *w*^*ndelT*^ and *st*^*1*^mutations (*w*^*ndelT*^*;;st*^*1*^) was generated. Male flies of triple mutant combinations were generated by crossing female flies of *w*^*ndelT*^*;;st*^*1*^ to male flies of either *cn*^*1*^, *cn*^*35k*^, *or cd*^*1*^. RNAi-mediated knockdown of the *white* gene was achieved with a line in which the GMR promoter drives the expression of white double stranded-hairpin RNA ([76], The Bloomington *Drosophila* Stock Center Line ID: BDSC_32067, called *wRNAi* here). RNAi-mediated knockdown of the *st* gene was achieved by driving the expression of transgene bearing an inverted repeat of *st* [26,77] with the *GMR-Gal4* line [78].

### 3OH-K and KYNA feeding

Animals were raised and maintained from embryonic stages onward on normal food supplemented with 3OH-K (1mg/ml of 3-Hydroxy-DL-kynurenine, H1771, Sigma Aldrich; dissolved in DMSO) or KYNA (1mg/ml of Kynurenic acid, K3375, Sigma Aldrich; dissolved in water).

### Experimental light conditions

Light conditions used here have been previously described [37]. Briefly, flies were reared in regular light conditions defined as 12 hours of light (approx. 900–1300 lux)/12 hours of darkness. For the light-induced degeneration paradigm, flies (2–4 days of age) were placed at 25°C for 7 days in an incubator dedicated for continuous, high intensity light exposure [79]. High intensity light was defined by 1200–1300 lux measured using an Extech 403125 Light ProbeMeter (Extech Instruments, USA) with the detector placed immediately adjacent to the vial and facing the nearest light source. To remove blue light in this setting (Fig 3), a customized box bounded by filters, which block blue light and face the light source in the incubator, was used. Light intensity was determined by measuring light counts using a USB spectrometer (Ocean Optics, USA).

### Histology and quantification from toluidine blue-stained sections

Histology and analyses of toluidine blue stained sections were carried out as previously described [37] and modified from [80]. To compare morphological aspects of degeneration across different conditions, two aspects of light-induced damage were quantified. These include

1. the number of identifiable ommatidia normalized to area. Light exposure causes holes (highlighted in yellow in Fig 2B) between adjacent ommatidia [also called lacunae [34]] and it results in the reduced density of ommatidia. Therefore the quantification of the density of ommatidia is a measure of the consequence of holes in the retinal tissue. For example, within the same area, a relatively undamaged retina (*w*^*ndelT*^) exhibits 11 identifiable ommatidia (S7 Fig) as opposed to only 7 identifiable ommatidia in a retina (*w*^*ndelT*^*;;st*^*1*^*/+*) with large holes (S7 Fig) following light exposure. For the quantification, number of ommatidia were normalized to unit area x 10.
2. the number of rhabdomeres per ommatidia. The number of identifiable rhabdomeres per ommatidia was counted to calculate the percent frequency of ommatidia with fewer than the normal complement of 7 rhabdomeres. This attribute is recorded as a proxy of photoreceptor (PRC) health. For example, in wild-type retinas 100% of ommatidia showed seven rhabdomeres/ommatidium in each section (S1A’ Fig), indicative of seven healthy PRCs. It should be noted that fewer than 7 rhabdomeres per ommatidia does not necessarily indicate cell death.

### Metabolite extraction, Mass spectrometry (MS) measurement

10 heads from age-matched (2–4 days old) male flies of genotypes indicated were pooled as one biological replicate and three such replicates were generated. Fly heads were frozen in liquid nitrogen and kept at -80°C until further use. Fly heads were homogenized using 0.5 mm zirconia beads for 2 x 5min in 100μl 80% methanol; 0.5% Formic acid and 50nM Internal standard Chloropropamide (Sigma-Aldrich Chemie GmbH). The mixture was centrifuged at 13.000g, and 10μl were analyzed. Mass spectrometric analysis was performed on a Q Exactive instrument (Thermo Fischer Scientific, Bremen, DE) equipped with a robotic nanoflow ion source TriVersa NanoMate (Advion BioSciences, Ithaca, USA) using nanoelectrospray chips with a diameter of 4.1 μm. The ion source was controlled by the Chipsoſt 8.3.1 soſtware (Advion BioSciences). Ionization voltage was + 0.96 kV in positive and − 0.96 kV in negative mode; backpressure was set at 1.25 psi in both modes. Samples were analyzed by polarity switching [81]. The temperature of the ion transfer capillary was 200°C; S-lens RF level was set to 50%. All samples were analyzed for 11 min. FT MS spectra were acquired within the range of m/z 150–350 from 0 min to 0.2 min in positive and within the range of m/z 150–350 from 6.2 min to 6.4 min in negative mode at the mass resolution of R m/z 200 = 140000; automated gain control (AGC) of 3×106 and with the maximal injection time of 3000 ms. t-SIM in positive (0.2 to 4.2 min) and negative (6.4 to 10.4 min) mode was acquired with R m/z 200 = 140000; automated gain control of 5 × 104; maximum injection time of 650 ms; isolation window of 20 Th and scan range of m/z 150 to 350 in positive and m/z 150 to 350 in negative mode, respectively. The inclusion list of masses targeted in t-SIM analyses started at m/z 150 in negative and m/z 150 in positive ion mode and other masses were computed by adding 10 Th increment (i.e. m/z 155, 165, 175, …) up to m/z 350. Quantification of all targeted molecules was performed by parallel reaction monitoring (PRM) FT MS/MS from 4.2 to 6 min in positive mode and from 10.4 to 11.0 min in negative mode. For PRM micro scans were set to 1, isolation window to 0.8 Da, normalized collision energy to 12.5%, AGC to 5 × 104 and maximum injection time to 650 ms. Representative spectra are shown in S8 Fig. All spectra were pre-processed using repetition rate filtering software PeakStrainer [82] and stitched together by an in-house developed script [83]. The targeted molecules were quantified by LipidXplorer soſtware [84].

### Western blotting

Three fly heads from age-matched flies were homogenized in 10 μl of 4x SDS-PAGE sample buffer (200 mM Tris-HCl pH 6.8, 20% Glycerol, 8% SDS, 0.04% Bromophenol blue, 400 mM DTT). After dilution with RIPA buffer (150 mM sodium chloride, 1% Triton X-100, 0.5% sodium deoxycholate, 0.1% SDS, 50 mM Tris pH 8), lysates were heated at 37°C for 30 min. Lysates equivalent to 2.5 heads were loaded and run on two parallel (15% acrylamide) gels, and proteins were transferred onto a membrane (Nitrocellulose Blotting Membrane 10600002; GE Healthcare Life Sciences; PA; USA). Primary antibodies were incubated overnight at 4°C and included mouse anti-3OH-K (1:1000; MA1-16616, AB_568420, clone P3UI, Thermo Fisher Scientific) and mouse anti-β-Tubulin E7 (1:1000; [http://dshb.biology.uiowa.edu/tubulin-beta-_2] deposited by M. McCutcheon/ S. Carroll to the from Developmental Studies Hybridoma Bank (DSHB), University of Iowa, USA). As secondary antibody IRDye 800CW goat anti-Mouse IgG (1,15,000; LI-COR Biotechnology; NE; US) was used for an 1 h incubation at room temperature. The dry membrane was imaged using a LI-COR Odyssey Sa Infrared Imaging System 9260-11P (LI-COR Biotechnology).

### Figure panel preparation and statistical analyses

Statistical power analyses was not conducted as part of experimental design to determine sample size. Sample sizes are evident in all dot plot graphs. For frequency distribution, sample sizes are indicated in the legend. All figure panels were assembled using Adobe Photoshop CS5.1 or Adobe Illustrator CS3 (Adobe Systems, USA). Statistical analyses and graphs were generated using GraphPad Prism (GraphPad Software, Inc, USA). All statistical comparisons are also provided in the S1 Dataset.

## Supporting information

S1 Fig The effect of pigmentation and shorter wavelength light on the extent of light-induced retinal damage in different genotypes. In A, C, E: Horizontal bars indicate the average number of ommatidia with identifiable rhabdomeres normalized to unit area. Each circle represents an individual count from 1 biological replicate. Number indicates the mean value for each condition. Only statistically significant differences between genotype pairs as revealed by ANOVA followed by Bonferroni’s post-hoc test at p<0.05, are indicated by * (or Unpaired t-test p<0.05, in C). In B, D, F: Percent frequency of ommatidia [± s.e.m.] displaying 1–7 rhabdomeres upon 7 days of continuous, high intensity light exposure. Numbers on top of each bar indicates the mean value of ommatidia displaying the full complement of 7 rhabdomeres. No statistically significant differences were observed between genotype pairs as revealed by ANOVA followed by Bonferroni’s post-hoc test at p<0.05 (or Unpaired t-test p<0.05, in D). In A-F, flies were exposed to high intensity, continuous light for 7 days. No damage is observed in a *w*^*+*^, pigmented background (A.B). Knocking-down *w* (*w-RNAi*) and removing of one copy of *st* (*w-RNAi;;st*^*1*^*/+*) results in retinal damage as compared to *w-RNAi* alone (C, D). Exposure to continuous, high intensity light lacking the short, blue wavelength component for 7 days (unfilled circles in E, unfilled bars in F) does not induce retinal damage of the indicated genotypes as compared to light with the short, blue wavelength component (filled circles in E and filled bars in F).
(TIF)

S2 Fig Retinal degeneration is not sex-specific and *cn*^*35K*^ modulates retinal degeneration to the same extent as *cn*^*1*^. **A**: Horizontal bars indicate the average number of ommatidia with identifiable rhabdomeres normalized to unit area for the genotypes indicated upon exposure of flies to continuous, high intensity white light for 7 days. Male genotype: *w*^*ndelT*^*/Y;;st*^*1*^*/+*, female genotype: *w*^*ndelT*^*/w*^*ndelT*^*;;st*^*1*^*/+*. Each dot represents an individual count from 1 biological replicate. “n.s.” indicates no statistically significant differences between genotype pairs revealed by Student’s t-test at p<0.05. **B**: Mean percent frequency of ommatidia displaying 1–7 rhabdomeres [± s.e.m.] upon exposure of flies to continuous, high intensity white light for 7 days. Male genotype: *w*^*ndelT*^*/Y;;st*^*1*^*/+*, female genotype: *w*^*ndelT*^*/w*^*ndelT*^*;;st*^*1*^*/+*. Numbers above bars indicate the mean percent value of ommatidia displaying the full complement of 7 rhabdomeres. “n.s.” indicates no statistically significant differences between genotype pairs revealed by Student’s t-test at p<0.05. **C:** Graph comparing the extent of retinal damage following exposure to continuous, high intensity white light for 7 days. Horizontal bars indicate the average number of ommatidia with identifiable rhabdomeres normalized to unit area. Each circle represents an individual count from 1 biological replicate. Number indicates the mean value for each condition. No statistically significant differences were recorded between *cn*^*1*^*/+* and *cn*^*35k*^*/+*. **D:** Percent frequency of ommatidia [± s.e.m.] displaying 1–7 rhabdomeres upon 7 days of continuous, high intensity, white light. Number on top of each bar indicates the mean value of ommatidia displaying the full complement of 7 rhabdomeres. Statistically significant differences, between genotype pairs as revealed by ANOVA followed by Bonferroni’s post-hoc test at p<0.05, are indicated by *. No statistically significant differences were recorded between *cn*^*1*^*/+* and *cn*^*35k*^*/+*.
(TIF)

S3 Fig Retinal degeneration in *w;;st* mutants is associated with altered pigment cell homeostasis and is not Rh1-dependent. **A-B**: Confocal images of 12μm sections of fly eyes raised under physiological light conditions (no retinal damage) and labelled with Phalloidin conjugated with a fluorescent dye (pseudocolored as magenta) and BODIPY (for lipid droplet accumulation, as a marker of altered retinal homeostasis (Liu, Zhang et al., 2015, Muliyil, Levet et al., 2020, Van Den Brink, Cubizolle et al., 2018), pseudocolored as yellow). There are more lipid droplets observed in *w*^*ndelT*^*;;st*^*1*^*/ st*^*1*^ as compared to *w*^*ndelT*^. **C-D**: Count masks derived in Fiji extracted from the BODIPY channel of the confocal images of longitudinal sections of fly eyes. **E**: Graph represents mean ± s.e.m. of number of lipid droplets quantified from images of longitudinal sections of the genotypes indicated labelled with BODIPY. Each dot represents a biological replicate and * indicates statistical significance for the pair as revealed by ANOVA followed by Bonferroni’s post-hoc test at p<0.05. **F**. Bars represent mean ± s.e.m. of the percent frequency of ommatidia displaying 1–7 rhabdomeres of ommatidia upon constant white light exposure for *w*^*ndelT*^*;;st*^*1*^*/+* flies raised with normal food (blue) or with carotenoid free food (patterned bars). Numbers above bars indicate the mean percent value for the subset of ommatidia displaying the full complement of 7 rhabdomeres.
(TIF)

S4 Fig Mass Spectrometric (MS) measurements of selected metabolites of the Kynurenine pathway (KP). **A, B, C, D:** Outline of relevant steps in the KP and brown pigment biosynthesis and genes analyzed in this study. The metabolite quantified and compared across genotypes in the corresponding graph (A’-D’) is highlighted in yellow. **A’-D’**: Graphs depicting the abundances of metabolites (highlighted in yellow in the corresponding pathway shown in A-D) from MS measurements (in arbitrary units along the X axis) of head extracts. Numbers on Y-axis correspond to the genotypes listed below. Each colored dot represents an individual biological replicate of 10 pooled heads. Statistical comparisons between genotype pairs are indicated with solid lines (red line compared to *+/+*, magenta line (A’) compared to *cn*^*1*^*/cn*^*1*^, orange line (B’) compared to *st*^*1*^*/st*^*1*^, grey line compared to *w*^*ndelT*^, blue line compared to *w*^*ndelT*^*;;st*^*1*^*/+*, brown line compared to *w*^*ndelT*^*;cn*^*1*^*/+*). Statistically significant differences between pairs of genotypes are indicated by * as revealed by ANOVA followed by Bonferroni’s post-hoc test at p<0.05.
(TIF)

S5 Fig Effect of dietary supplementation of 3OH-K on eye color and the amount of protein-bound 3OH-K. **A:** Bright-field images of *cn*^*1*^*/cn*^*1*^ flies raised on control food (+DMSO) or on food supplemented with 1mg/ml 3OH-K (+3OH-K). Control food-fed flies exhibit bright red eye color indicative of incomplete brown pigment biosynthesis (as expected of *cn* perturbation). Dietary 3OH-K reverts the eye color to dark red, indicative of proper brown pigment biogenesis. **B-B’:** Western blots of protein extracts from heads of wildtype adult flies (*+/+*) raised on control food (+DMSO), or on food supplemented with either 1mg/ml KYNA or 1mg/ml 3OH-K. Blots were tested with antibodies against 3OH-K (B) and against β-Tubulin as loading control (B’) run on parallel gels. Positions of standard molecular weight markers are indicated by arrowheads. Grey dotted lines outline the lanes of each sample.
(TIF)

S6 Fig *st* mutants exhibit altered oxidative stress signaling and *w;;st* mutants exhibit more intense staining of an indicator for reactive oxygen species (ROS). **A:** Bars represent mean fold-change in head mRNA levels normalized with mRNA of a reference gene (*gapdh1*). A panel of 10 genes annotated as oxidative stress response genes is shown on the X-axis. Fold change in *st*^*1*^*/st*^*1*^ (orange) was calculated with ΔΔCt method with respect to mRNA levels of control/wild-type (*+/+;* red), which were set to 1. Experiment was done in triplicates and * indicate significant differences in fold-change calculated with a Student’s t-test, p<0.05. **B-C:** Images of whole tissue preparations of adult *w*^*ndelT*^ (B) and *w*^*ndelT*^*;;st*^*1*^*/+* (C) eyes labelled with Dihydroethidium (DHE), an indicator for reactive oxygen species (ROS) (Robinson, Janes et al., 2006). DHE staining intensity appears to be higher in *w*^*ndelT*^*;;st*^*1*^*/+* (C) as compared to *w*^*ndelT*^ (B). Distal and proximal ends of the retina are indicated and one ommatidium is outlined with a dotted line.
(TIF)

S7 Fig The influence of light exposure on retinal histology. **A-B:** Examples of images of toluidine-blue stained 1μm thin sections of fly eyes of *w*^*ndelT*^ (A) and *w*^*ndelT*^*;;st*^*1*^*/+* (B), following exposure to high intensity continuous white light for 7 days. Qualitatively varying degrees of light-induced damage are observed including holes (lacunae) (highlighted in yellow in B), ommatidia with less than 7 rhabdomeres (red dotted circles), and the presence of apoptotic debris (intensely stained regions; arrows in B). Scale bar as indicated in A. These phenotypes appear more pronounced in B than in A. Of these attributes, we quantified the following: (i) the consequence of lacunae formation by estimating ommatidial density or the number of identifiable ommatidia per unit area (11 in A, 7 in B). (ii) the status of photoreceptor (PRC) health by estimating the frequency of ommatidia with 7 or fewer than 7 rhabdomeres.
(TIF)

S8 Fig Shotgun determination of the KP compounds performed on a Q-Exactive MS instrument. A-D’ are representative spectra for standards (A-C) and for metabolites extracted from fly head samples (A’-D’). Red colored spectra indicate the specific fragment corresponding to the precursor ion following fragmentation. **A, A’**: MS/MS product ion spectrum of the [M-H]^-^ ion of Kynurenic acid (m/z = 188.03). The product ion (m/z 144.04) is obtained from loss of CO_2_. **B, B’**: MS/MS product ion spectrum of the [M+H]^+^ ion of Kynurenine (m/z = 209.09). Different product ions obtained from the precursor are identified with m/z: 192.07; 174.05; 146.06; 136.08. **C, C’**: MS/MS product ion spectrum of the [M+H]^+^ ion of 3-hydroxy Kynurenine (m/z = 225.09). Different product ions obtained from the precursor are identified with m/z: 208.06; 190.05; 162.05; 152.07; 110.06. **D’**: MS/MS product ion spectrum of the [M-H]^-^ ion of Xanthurenic acid (m/z 204.03). The product ion (m/z = 172.04) is obtained from loss of 2x H_2_O.
(TIF)

S1 Dataset Supporting Dataset. (XLSX)

S1 Methods Supplementary methods. (DOCX)
