# Supplementary figures and images for: Modulating the Kynurenine pathway or sequestering toxic 3-hydroxykynurenine protects the retina from light-induced damage in Drosophila
Source: PLoS Genet. 2023 Mar 23;19(3):e1010644. doi: 10.1371/journal.pgen.1010644 (PMC10035932; doi:10.1371/journal.pgen.1010644)

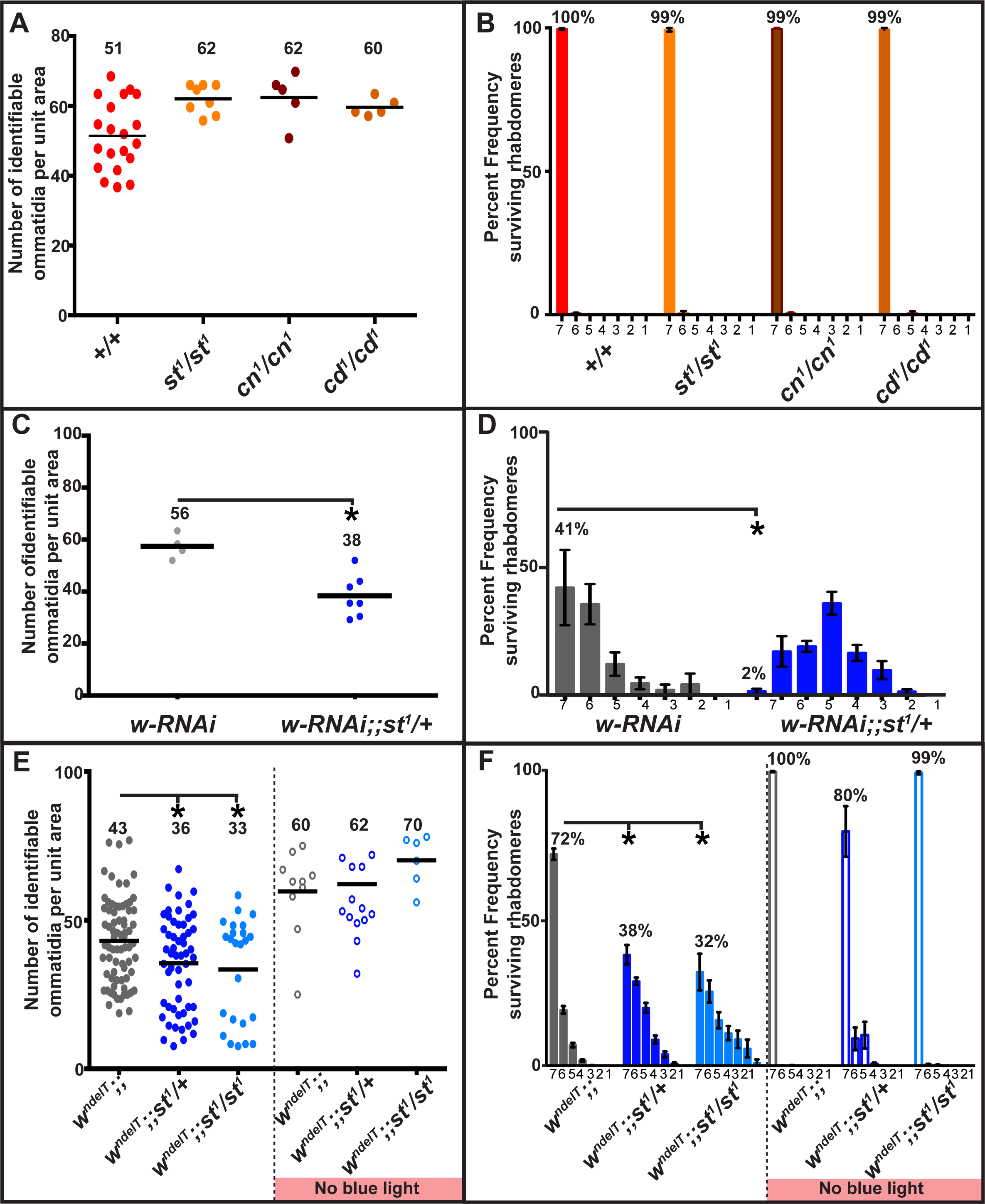

Supplement: S1 Fig — In A, C, E: Horizontal bars indicate the average number of ommatidia with identifiable rhabdomeres normalized to unit area. Each circle represents an individual count from 1 biological replicate. Number indicates the mean value for each condition. Only statistically significant differences between genotype pairs as revealed by ANOVA followed by Bonferroni’s post-hoc test at p<0.05, are indicated by * (or Unpaired t-test p<0.05, in C). In B, D, F: Percent frequency of ommatidia [± s.e.m.] displaying 1–7 rhabdomeres upon 7 days of continuous, high intensity light exposure. Numbers on top of each bar indicates the mean value of ommatidia displaying the full complement of 7 rhabdomeres. No statistically significant differences were observed between genotype pairs as revealed by ANOVA followed by Bonferroni’s post-hoc test at p<0.05 (or Unpaired t-test p<0.05, in D). In A-F, flies were exposed to high intensity, continuous light for 7 days. No damage is observed in a w+, pigmented background (A.B). Knocking-down w (w-RNAi) and removing of one copy of st (w-RNAi;;st1/+) results in retinal damage as compared to w-RNAi alone (C, D). Exposure to continuous, high intensity light lacking the short, blue wavelength component for 7 days (unfilled circles in E, unfilled bars in F) does not induce retinal damage of the indicated genotypes as compared to light with the short, blue wavelength component (filled circles in E and filled bars in F). (TIF) [file pgen.1010644.s001.tif]

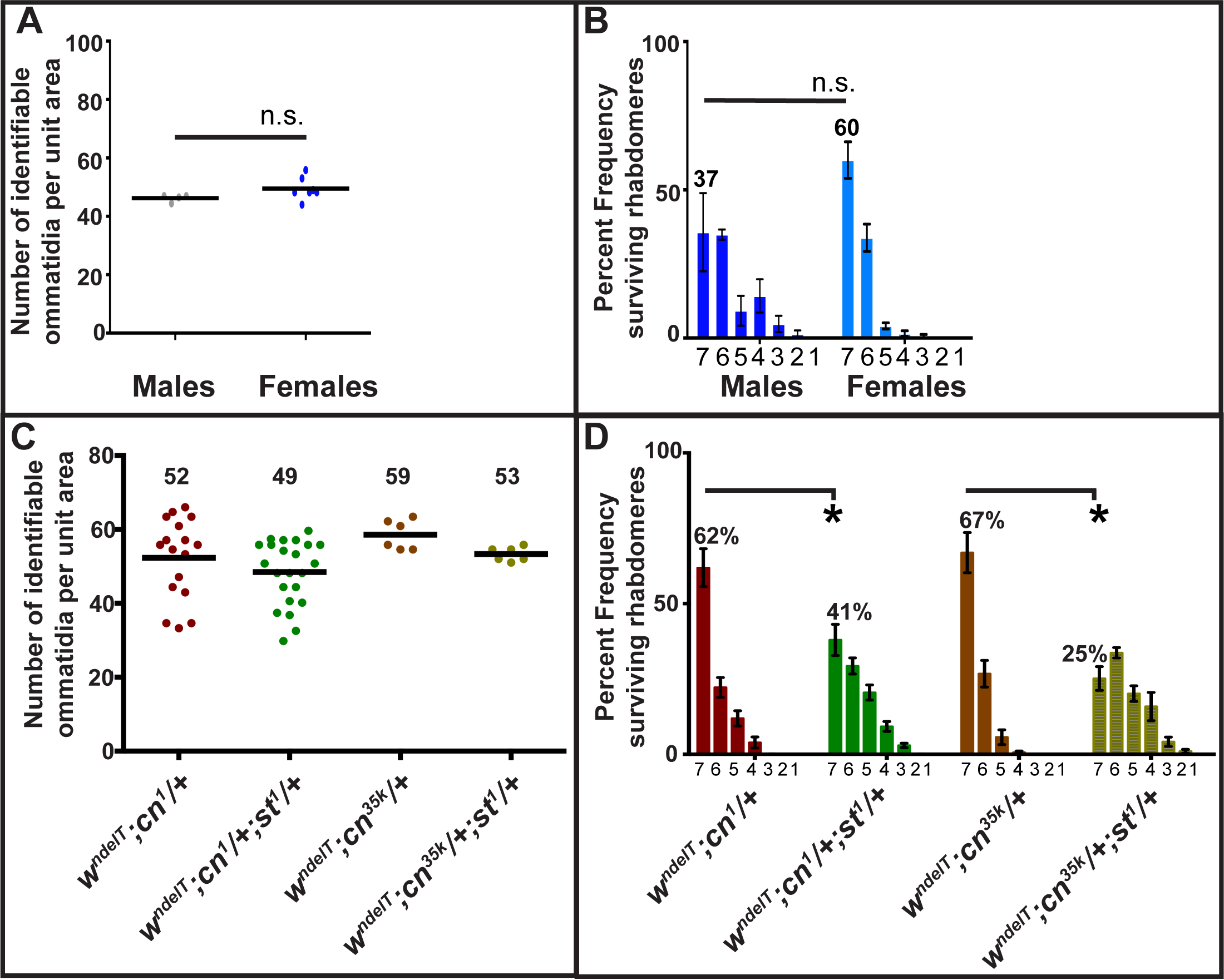

Supplement: S2 Fig — A: Horizontal bars indicate the average number of ommatidia with identifiable rhabdomeres normalized to unit area for the genotypes indicated upon exposure of flies to continuous, high intensity white light for 7 days. Male genotype: wndelT/Y;;st1/+, female genotype: wndelT/wndelT;;st1/+. Each dot represents an individual count from 1 biological replicate. “n.s.” indicates no statistically significant differences between genotype pairs revealed by Student’s t-test at p<0.05. B: Mean percent frequency of ommatidia displaying 1–7 rhabdomeres [± s.e.m.] upon exposure of flies to continuous, high intensity white light for 7 days. Male genotype: wndelT/Y;;st1/+, female genotype: wndelT/wndelT;;st1/+. Numbers above bars indicate the mean percent value of ommatidia displaying the full complement of 7 rhabdomeres. “n.s.” indicates no statistically significant differences between genotype pairs revealed by Student’s t-test at p<0.05. C: Graph comparing the extent of retinal damage following exposure to continuous, high intensity white light for 7 days. Horizontal bars indicate the average number of ommatidia with identifiable rhabdomeres normalized to unit area. Each circle represents an individual count from 1 biological replicate. Number indicates the mean value for each condition. No statistically significant differences were recorded between cn1/+ and cn35k/+. D: Percent frequency of ommatidia [± s.e.m.] displaying 1–7 rhabdomeres upon 7 days of continuous, high intensity, white light. Number on top of each bar indicates the mean value of ommatidia displaying the full complement of 7 rhabdomeres. Statistically significant differences, between genotype pairs as revealed by ANOVA followed by Bonferroni’s post-hoc test at p<0.05, are indicated by *. No statistically significant differences were recorded between cn1/+ and cn35k/+. (TIF) [file pgen.1010644.s002.tif]

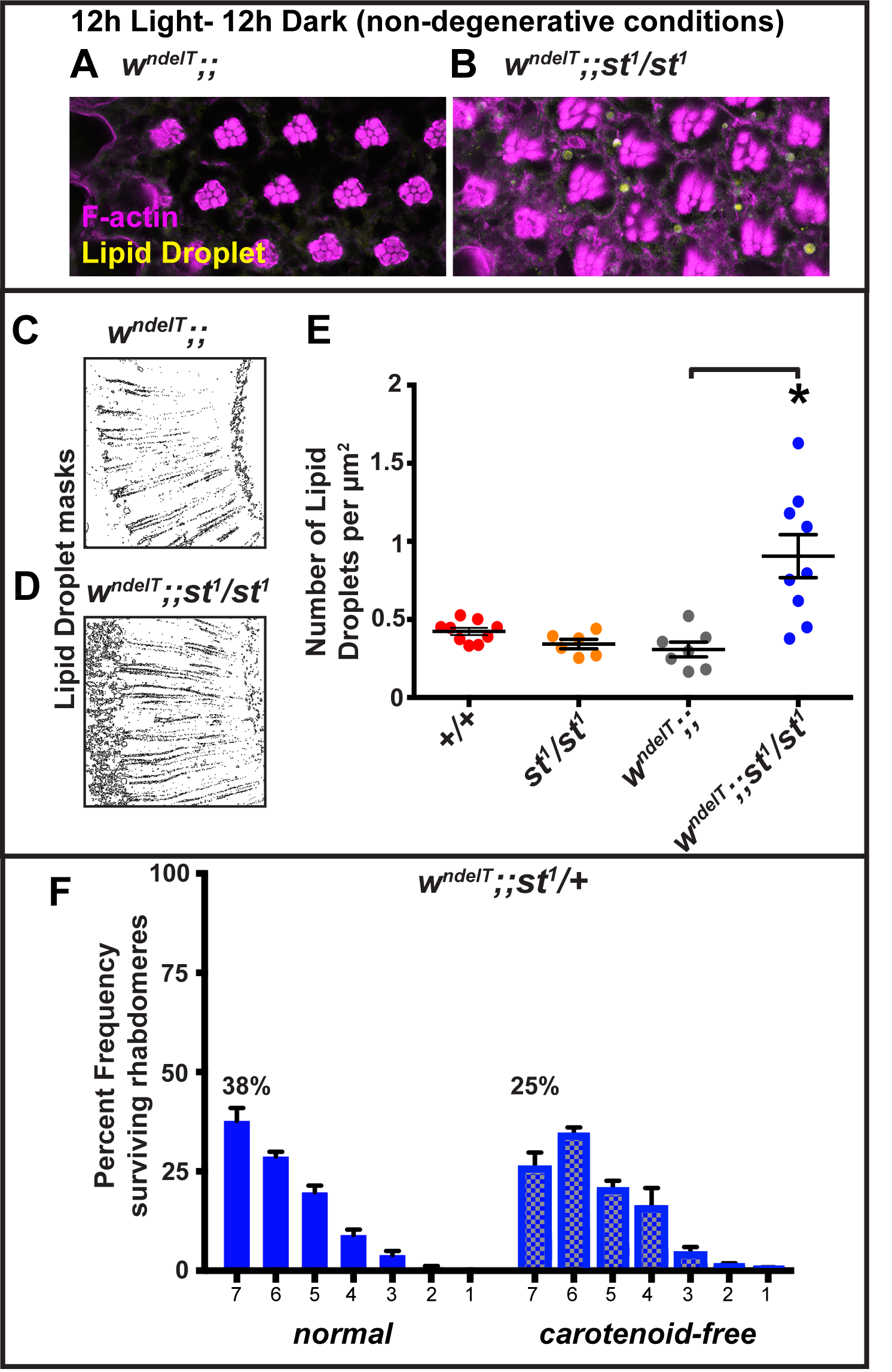

Supplement: S3 Fig — A-B: Confocal images of 12μm sections of fly eyes raised under physiological light conditions (no retinal damage) and labelled with Phalloidin conjugated with a fluorescent dye (pseudocolored as magenta) and BODIPY (for lipid droplet accumulation, as a marker of altered retinal homeostasis (Liu, Zhang et al., 2015, Muliyil, Levet et al., 2020, Van Den Brink, Cubizolle et al., 2018), pseudocolored as yellow). There are more lipid droplets observed in wndelT;;st1/ st1 as compared to wndelT. C-D: Count masks derived in Fiji extracted from the BODIPY channel of the confocal images of longitudinal sections of fly eyes. E: Graph represents mean ± s.e.m. of number of lipid droplets quantified from images of longitudinal sections of the genotypes indicated labelled with BODIPY. Each dot represents a biological replicate and * indicates statistical significance for the pair as revealed by ANOVA followed by Bonferroni’s post-hoc test at p<0.05. F. Bars represent mean ± s.e.m. of the percent frequency of ommatidia displaying 1–7 rhabdomeres of ommatidia upon constant white light exposure for wndelT;;st1/+ flies raised with normal food (blue) or with carotenoid free food (patterned bars). Numbers above bars indicate the mean percent value for the subset of ommatidia displaying the full complement of 7 rhabdomeres. (TIF) [file pgen.1010644.s003.tif]

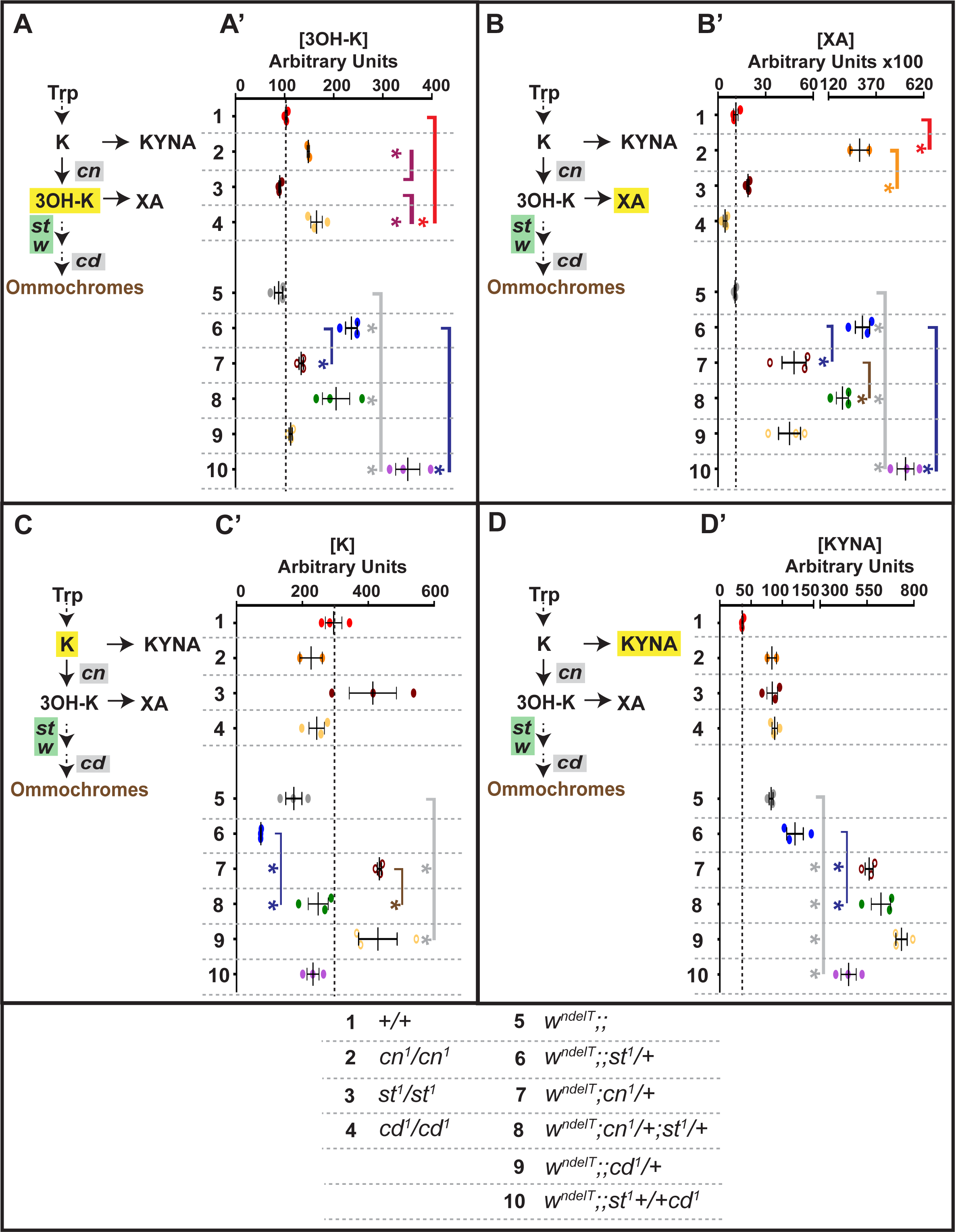

Supplement: S4 Fig — A, B, C, D: Outline of relevant steps in the KP and brown pigment biosynthesis and genes analyzed in this study. The metabolite quantified and compared across genotypes in the corresponding graph (A’-D’) is highlighted in yellow. A’-D’: Graphs depicting the abundances of metabolites (highlighted in yellow in the corresponding pathway shown in A-D) from MS measurements (in arbitrary units along the X axis) of head extracts. Numbers on Y-axis correspond to the genotypes listed below. Each colored dot represents an individual biological replicate of 10 pooled heads. Statistical comparisons between genotype pairs are indicated with solid lines (red line compared to +/+, magenta line (A’) compared to cn1/cn1, orange line (B’) compared to st1/st1, grey line compared to wndelT, blue line compared to wndelT;;st1/+, brown line compared to wndelT;cn1/+). Statistically significant differences between pairs of genotypes are indicated by * as revealed by ANOVA followed by Bonferroni’s post-hoc test at p<0.05. (TIF) [file pgen.1010644.s004.tif]

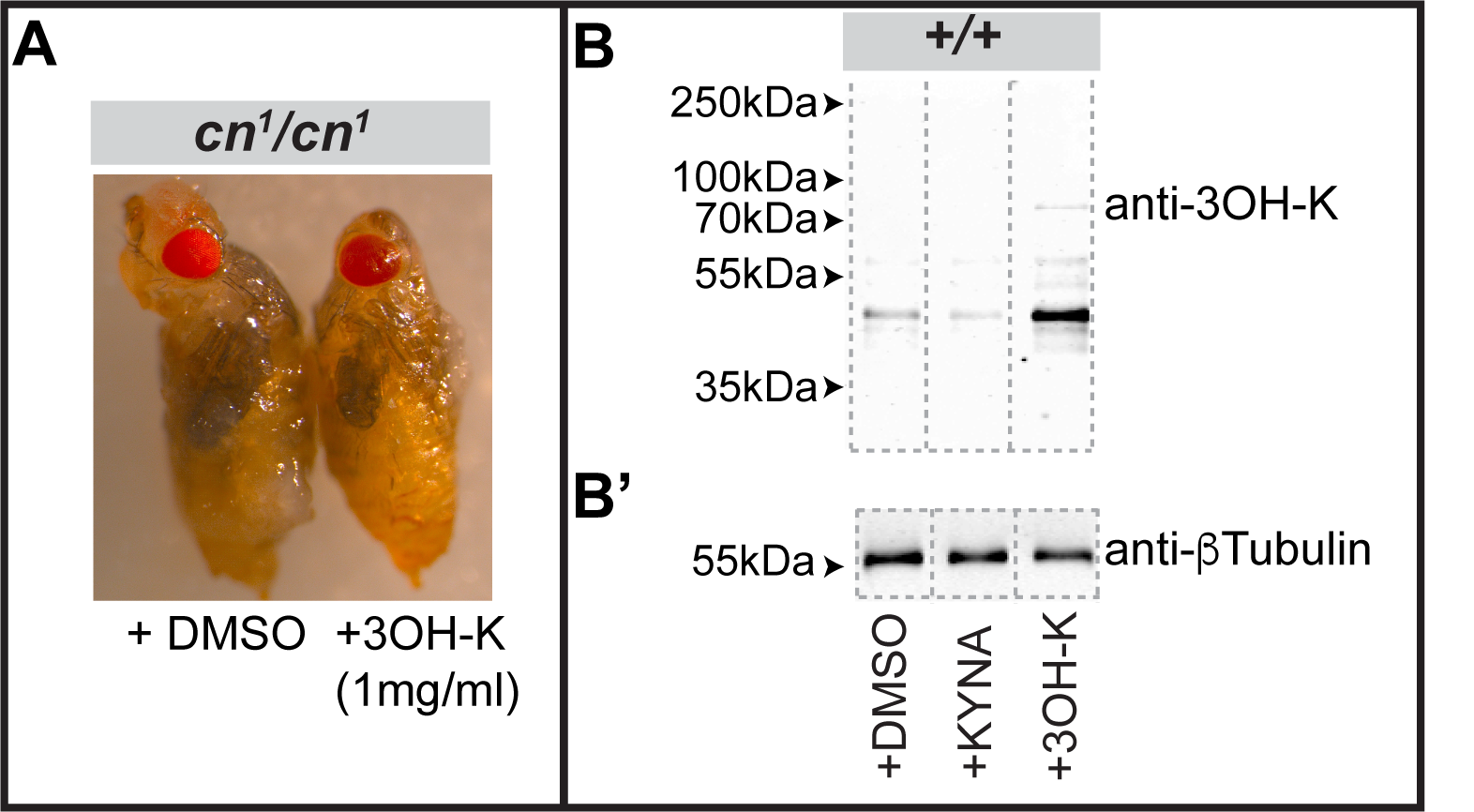

Supplement: S5 Fig — A: Bright-field images of cn1/cn1 flies raised on control food (+DMSO) or on food supplemented with 1mg/ml 3OH-K (+3OH-K). Control food-fed flies exhibit bright red eye color indicative of incomplete brown pigment biosynthesis (as expected of cn perturbation). Dietary 3OH-K reverts the eye color to dark red, indicative of proper brown pigment biogenesis. B-B’: Western blots of protein extracts from heads of wildtype adult flies (+/+) raised on control food (+DMSO), or on food supplemented with either 1mg/ml KYNA or 1mg/ml 3OH-K. Blots were tested with antibodies against 3OH-K (B) and against β-Tubulin as loading control (B’) run on parallel gels. Positions of standard molecular weight markers are indicated by arrowheads. Grey dotted lines outline the lanes of each sample. (TIF) [file pgen.1010644.s005.tif]

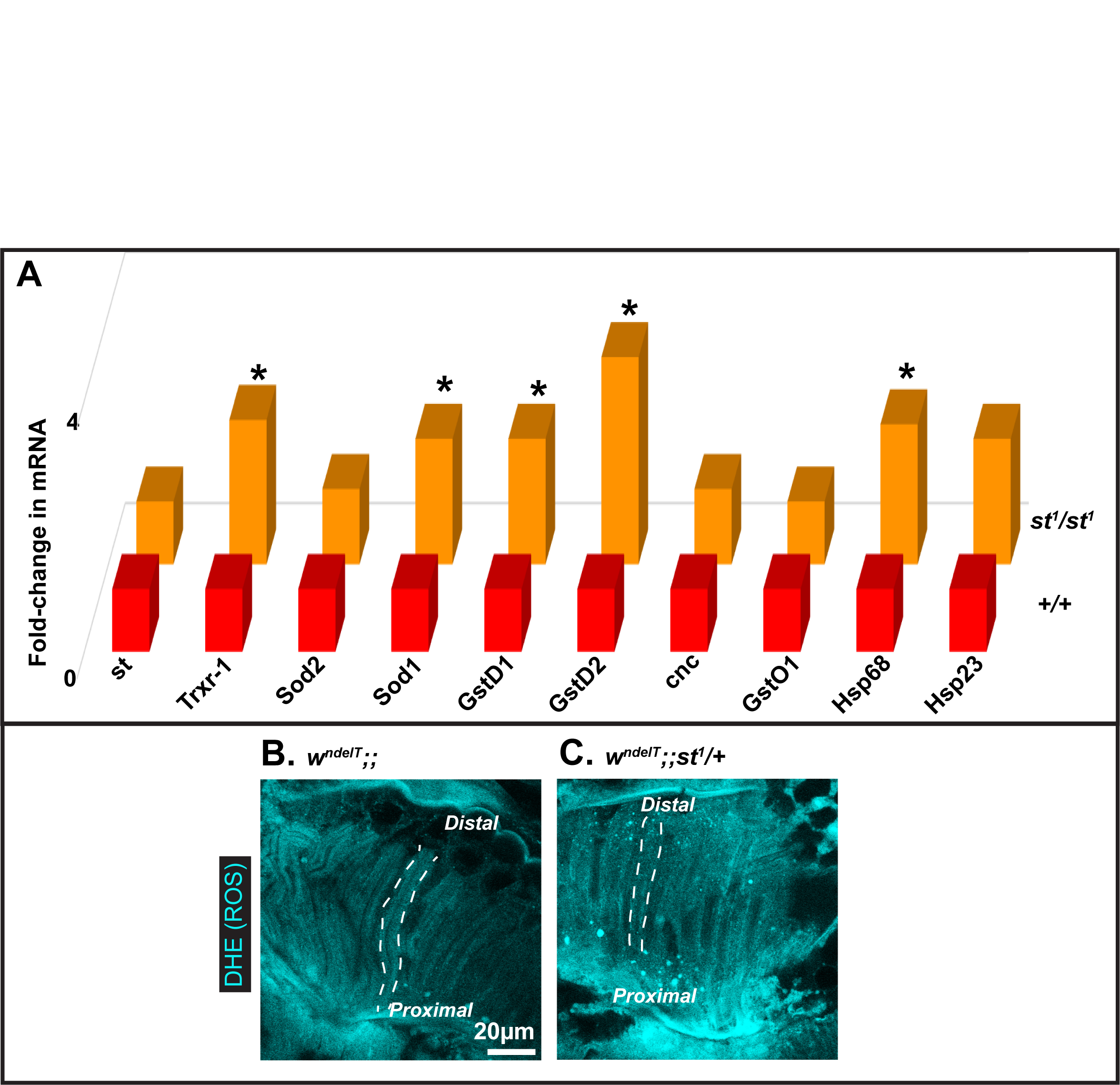

Supplement: S6 Fig — A: Bars represent mean fold-change in head mRNA levels normalized with mRNA of a reference gene (gapdh1). A panel of 10 genes annotated as oxidative stress response genes is shown on the X-axis. Fold change in st1/st1 (orange) was calculated with ΔΔCt method with respect to mRNA levels of control/wild-type (+/+; red), which were set to 1. Experiment was done in triplicates and * indicate significant differences in fold-change calculated with a Student’s t-test, p<0.05. B-C: Images of whole tissue preparations of adult wndelT (B) and wndelT;;st1/+ (C) eyes labelled with Dihydroethidium (DHE), an indicator for reactive oxygen species (ROS) (Robinson, Janes et al., 2006). DHE staining intensity appears to be higher in wndelT;;st1/+ (C) as compared to wndelT (B). Distal and proximal ends of the retina are indicated and one ommatidium is outlined with a dotted line. (TIF) [file pgen.1010644.s006.tif]

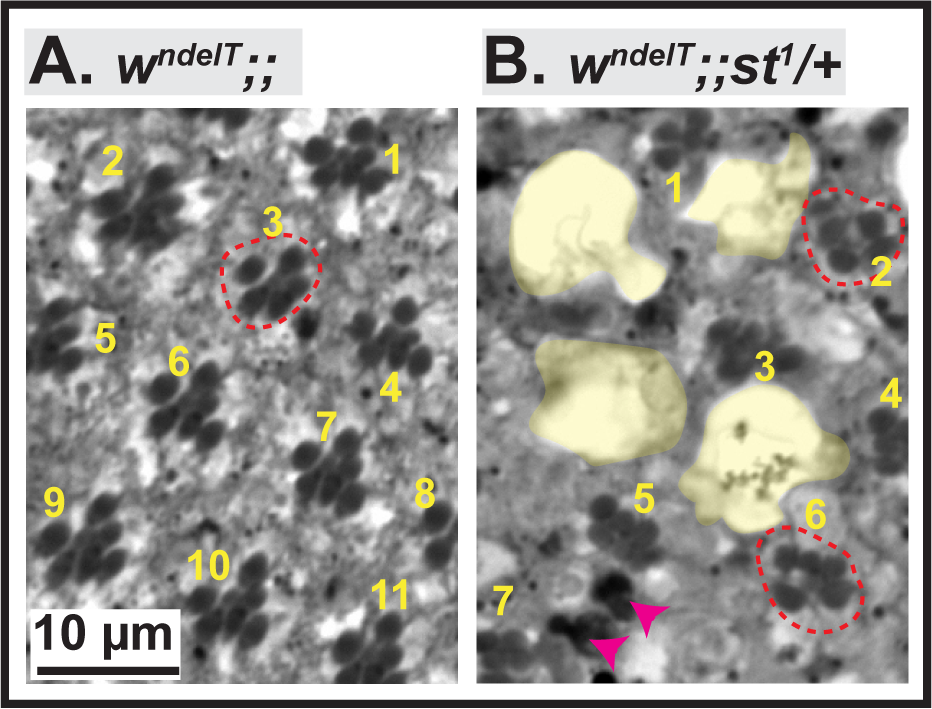

Supplement: S7 Fig — A-B: Examples of images of toluidine-blue stained 1μm thin sections of fly eyes of wndelT (A) and wndelT;;st1/+ (B), following exposure to high intensity continuous white light for 7 days. Qualitatively varying degrees of light-induced damage are observed including holes (lacunae) (highlighted in yellow in B), ommatidia with less than 7 rhabdomeres (red dotted circles), and the presence of apoptotic debris (intensely stained regions; arrows in B). Scale bar as indicated in A. These phenotypes appear more pronounced in B than in A. Of these attributes, we quantified the following: (i) the consequence of lacunae formation by estimating ommatidial density or the number of identifiable ommatidia per unit area (11 in A, 7 in B). (ii) the status of photoreceptor (PRC) health by estimating the frequency of ommatidia with 7 or fewer than 7 rhabdomeres. (TIF) [file pgen.1010644.s007.tif]

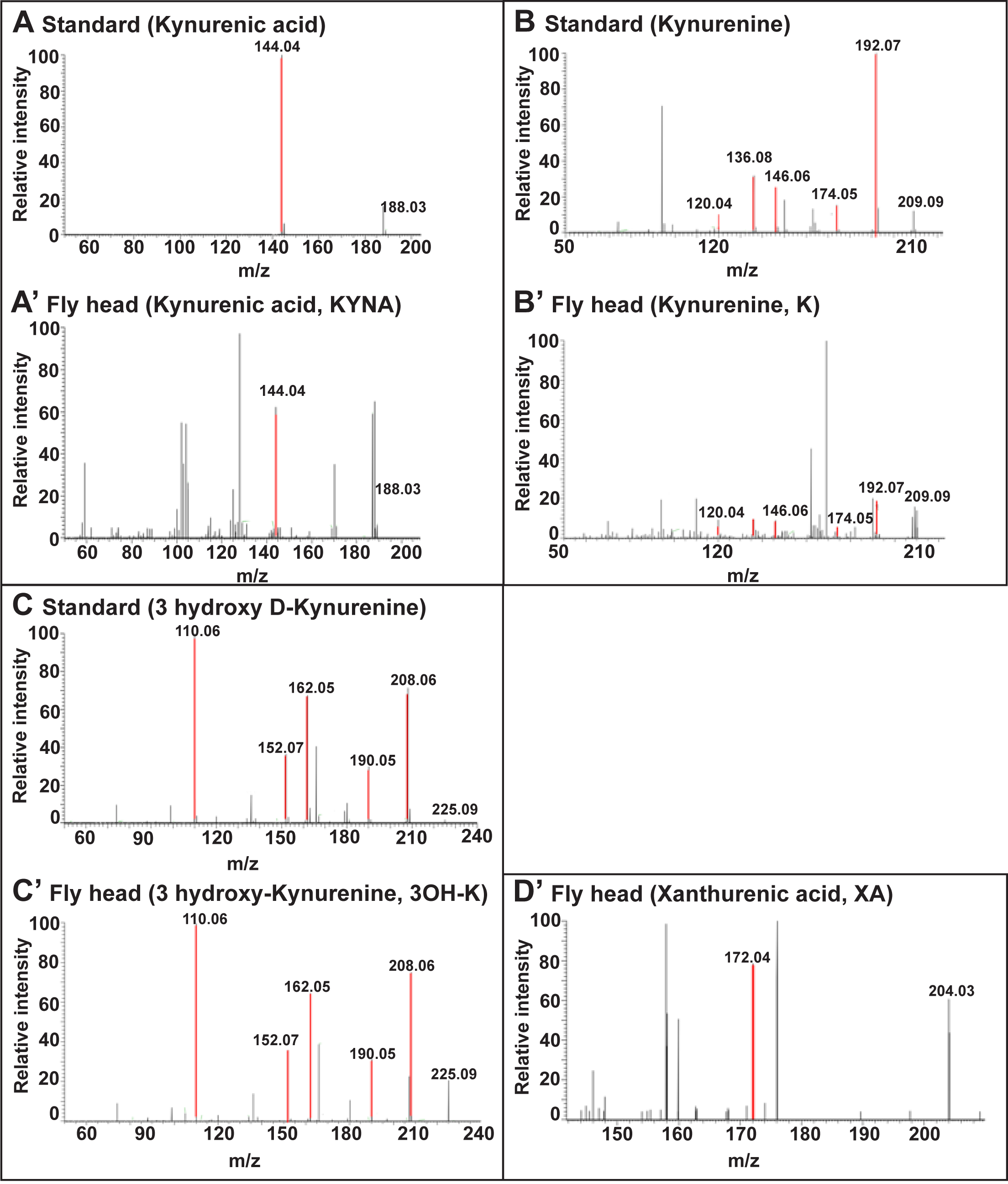

Supplement: S8 Fig — A-D’ are representative spectra for standards (A-C) and for metabolites extracted from fly head samples (A’-D’). Red colored spectra indicate the specific fragment corresponding to the precursor ion following fragmentation. A, A’: MS/MS product ion spectrum of the [M-H]- ion of Kynurenic acid (m/z = 188.03). The product ion (m/z 144.04) is obtained from loss of CO2. B, B’: MS/MS product ion spectrum of the [M+H]+ ion of Kynurenine (m/z = 209.09). Different product ions obtained from the precursor are identified with m/z: 192.07; 174.05; 146.06; 136.08. C, C’: MS/MS product ion spectrum of the [M+H]+ ion of 3-hydroxy Kynurenine (m/z = 225.09). Different product ions obtained from the precursor are identified with m/z: 208.06; 190.05; 162.05; 152.07; 110.06. D’: MS/MS product ion spectrum of the [M-H]- ion of Xanthurenic acid (m/z 204.03). The product ion (m/z = 172.04) is obtained from loss of 2x H2O. (TIF) [file pgen.1010644.s008.tif]
