# Supplementary material for: Modulating the Kynurenine pathway or sequestering toxic 3-hydroxykynurenine protects the retina from light-induced damage in Drosophila
Source: PLoS Genet. 2023 Mar 23;19(3):e1010644. doi: 10.1371/journal.pgen.1010644 (PMC10035932; doi:10.1371/journal.pgen.1010644)
Supplement: S1 Methods — (DOCX) [file pgen.1010644.s010.docx]

**Supplementary Methods**

**Carotenoid-depleted diet** was prepared as described in [1] and flies were raised on this diet from embryos to adulthood.

**Detection of reactive oxygen species (ROS), lipid droplets in eyes and imaging**

Eyes were dissected and processed for whole mount staining or for cryosectioning as described in [2, 3]. Superoxide radicals (ROS) were detected using Dihydroethidium (DHE [4, 5], Thermo Fisher Scientific). Briefly, adult eyes were dissected in a phosphate buffered solution, incubated in DHE (30µM), fixed in 4% formaldehyde for 5 minutes and immediately imaged. Lipid droplets in eye sections were visualized with a protocol adapted from [6] using 1µg/ml of BODIPY™ 500/510 C_1_, C_12_ (ThermoFisher Scientific) for an overnight incubation at 4ºC. Rhabdomeres were visualized using Alexa-Fluor-conjugated–phalloidin (Thermo Fisher Scientific) following an incubation of 1 hour at room temperature. Sections were imaged with an Olympus Fluoview 1000 confocal microscope using an Olympus UPlanS Apochromat 60x Oil objective (N.A. =1.35). They were subsequently visualized in Fiji [7], corrected for brightness and contrast, or analyzed for lipid droplet counts after applying a similar threshold on all images.

**Real-Time Quantitative Reverse Transcription Polymerase Chain Reaction (qRT-PCR) analyses**

RNA extraction, cDNA generation, qPCR analyses were performed as

described in [8]. Primers were designed using Primer-BLAST [9] or using precomputed primer designs found in FlyPrimerBank [10]. The primers pairs utilized in this study are as follows:

*st* (TCTAATGGGCTCGAGTGGC and CTGATGCGATGCATGAAGGG)

*Trxr-1*(AAAAGCAGGATGATGGCAAG and CCACAGAACGGTGTCGTAAA),

*Sod2* (TTTCGCAAACTGCAAGCCTG and TGATCTCCCGGCAGATGATAG),

*Sod1* (GGACCGCACTTCAATCCGTA and TGGAGTCGGTGATGTTGACC),

*GstD1* (CGCGCCATCCAGGTGTATTT and CTGGTACAGCGTTCCCATGT),

*GstD2* (ACATTGCCATCCTGTCCACTG and TCCTGGAGTCACCTTCTTGGC),

*cnc* (TCGGAGATGACGAGGAGGAGAGT and GCATTGATGATCGCCTCCTGGT),

*GstO1* (AGCTGTATTCGATGCGCTTT and GGTTTGTCGCGAAGATTGAT),

*Hsp68* (CCATCATGACCAAGATGCAC and ACGGTGGGACCCTTATAACC),

*Hsp23* (GTGTCGAAAATCGGAAAGGA and ACGGAGTTGTCCTGCACTTT),

*Gapdh1* (TAAATTCGACTCGACTCACGGT and CTCCACCACATACTCGGCTC)

**References**

1. Pocha SM, Shevchenko A, Knust E. Crumbs regulates rhodopsin transport by interacting with and stabilizing myosin V. J Cell Biol. 2011;195(5):827-38. Epub 2011/11/23. doi: 10.1083/jcb.201105144. PubMed PMID: 22105348; PubMed Central PMCID: PMCPMC3257572.

2. Hebbar S, Lehmann M, Behrens S, Halsig C, Leng W, Yuan M, et al. Mutations in the splicing regulator Prp31 lead to retinal degeneration in *Drosophila*. Biol Open. 2021;10(1). Epub 2021/01/27. doi: 10.1242/bio.052332. PubMed PMID: 33495354; PubMed Central PMCID: PMCPMC7860132.

3. Spannl S, Kumichel A, Hebbar S, Kapp K, Gonzalez-Gaitan M, Winkler S, et al. The Crumbs_C isoform of *Drosophila* shows tissue- and stage-specific expression and prevents light-dependent retinal degeneration. Biol Open. 2017;6(2):165-75. Epub 2017/02/17. doi: 10.1242/bio.020040. PubMed PMID: 28202468; PubMed Central PMCID: PMCPMC5312091.

4. Owusu-Ansah E, Banerjee U. Reactive oxygen species prime Drosophila haematopoietic progenitors for differentiation. Nature. 2009;461(7263):537-41. Epub 2009/09/04. doi: 10.1038/nature08313. PubMed PMID: 19727075; PubMed Central PMCID: PMCPMC4380287.

5. Robinson KM, Janes MS, Pehar M, Monette JS, Ross MF, Hagen TM, et al. Selective fluorescent imaging of superoxide in vivo using ethidium-based probes. Proc Natl Acad Sci U S A. 2006;103(41):15038-43. Epub 2006/10/04. doi: 10.1073/pnas.0601945103. PubMed PMID: 17015830; PubMed Central PMCID: PMCPMC1586181.

6. Van Den Brink DM, Cubizolle A, Chatelain G, Davoust N, Girard V, Johansen S, et al. Physiological and pathological roles of FATP-mediated lipid droplets in *Drosophila* and mice retina. PLoS Genet. 2018;14(9):e1007627. Epub 2018/09/11. doi: 10.1371/journal.pgen.1007627. PubMed PMID: 30199545; PubMed Central PMCID: PMCPMC6147681.

7. Schindelin J, Arganda-Carreras I, Frise E, Kaynig V, Longair M, Pietzsch T, et al. Fiji: an open-source platform for biological-image analysis. Nat Methods. 2012;9(7):676-82. Epub 2012/06/30. doi: 10.1038/nmeth.2019. PubMed PMID: 22743772; PubMed Central PMCID: PMCPMC3855844.

8. Hebbar S, Schuhmann K, Shevchenko A, Knust E. Hydroxylated sphingolipid biosynthesis regulates photoreceptor apical domain morphogenesis. J Cell Biol. 2020;219(12). Epub 2020/10/14. doi: 10.1083/jcb.201911100. PubMed PMID: 33048164; PubMed Central PMCID: PMCPMC7557679.

9. Ye J, Coulouris G, Zaretskaya I, Cutcutache I, Rozen S, Madden TL. Primer-BLAST: a tool to design target-specific primers for polymerase chain reaction. BMC Bioinformatics. 2012;13:134. Epub 2012/06/20. doi: 10.1186/1471-2105-13-134. PubMed PMID: 22708584; PubMed Central PMCID: PMCPMC3412702.

10. Hu Y, Sopko R, Foos M, Kelley C, Flockhart I, Ammeux N, et al. FlyPrimerBank: an online database for Drosophila melanogaster gene expression analysis and knockdown evaluation of RNAi reagents. G3 (Bethesda). 2013;3(9):1607-16. Epub 2013/07/31. doi: 10.1534/g3.113.007021. PubMed PMID: 23893746; PubMed Central PMCID: PMCPMC3755921.
